# BS-clock, advancing epigenetic age prediction with high-resolution DNA methylation bisulfite sequencing data

**DOI:** 10.1093/bioinformatics/btae656

**Published:** 2024-11-05

**Authors:** Congcong Hu, Yunxiao Li, Longhui Li, Naiqian Zhang, Xiaoqi Zheng

**Affiliations:** Department of Mathematics, Shanghai Normal University, Shanghai 200234, China; Department of Mathematics, Shanghai Normal University, Shanghai 200234, China; The Guangxi Key Laboratory of Intelligent Precision Medicine, Nanning, Guangxi Zhuang Autonomous Region 530028, China; Department of Mathematics, Shanghai Normal University, Shanghai 200234, China; School of Mathematics and Statistics, Shandong University, Weihai 264209, China; Center for Single-Cell Omics, School of Public Health, Shanghai Jiao Tong University School of Medicine, Shanghai 200025, China

## Abstract

**Motivation:**

DNA methylation patterns provide precise and accurate estimates of biological age due to their robustness and predictable changes associated with aging processes. Although several methylation aging clocks have been developed in recent years, they are primarily designed for DNA methylation array data, which has limited CpG coverage and detection sensitivity compared to bisulfite sequencing data.

**Results:**

Here, we present BS-clock, a novel DNA methylation clock for human aging based on bisulfite sequencing data. Using BS-seq data from 529 samples retrieved from four tissues, our BS-clock achieves higher correlations with chronological age in multiple tissue types compared to existing array-based clocks. Our study revealed age-dependent aging rates across different age stages and disease conditions, and overall low cross-tissue prediction capability by applying the model trained on one tissue type to others. In summary, BS-clock overcomes limitations of array-based techniques, offering genome-wide CpG site coverage and more robust and accurate aging quantification. This research paves the way for advanced epigenetic studies of aging and holds promise for developing targeted interventions to promote healthy aging.

**Availability and implementation:**

All analysis codes for reproducing the results of the study are publicly available at https://github.com/hucongcong97/BS-clock.

## 1 Introduction

Aging is a fundamental biological process closely linked to various chronic diseases and health issues (Partridge *et al.* 2018, Campisi *et al.* 2019, López-Otín *et al.* 2023). Understanding the mechanisms of aging and developing anti-aging interventions are crucial for extending healthy lifespan and improving the quality of life for the elderly (Morrisey *et al.* 1998). To assess and comprehend the aging process, researchers have developed various aging clocks, using data from DNA methylation (Field *et al.* 2018, Horvath and Raj 2018, Bell *et al.* 2019), gene expression (Lu *et al.* 2023, Zhu *et al.* 2023), protein and metabolite profiles (Wu *et al.* 2021, Li *et al.* 2023), or telomere length (Fyhrquist and Saijonmaa 2012, Jylhävä *et al.* 2017). These clocks evaluate biomarkers through multiple types of data to predict biological age, providing a powerful tool to quantify age-related health outcomes. Among these data types, DNA methylation patterns provide the highest precision and accurate estimates of biological age due to their robustness and predictable changes over multiple fundamental biological processes associated with aging, such as cellular senescence (Crouch *et al.* 2022), DNA repair (Schär and Fritsch 2011), and oxidative stress (Yara *et al.* 2015, Sundar *et al.* 2022). More importantly, epigenetic changes in DNA methylation biomarkers are reversible to environmental and lifestyle factors, making the estimation of DNA methylation age potentially useful for identifying or validating anti-aging interventions (Nwanaji-Enwerem and Colicino 2020, Fiorito *et al.* 2021).

In the past few years, a number of DNA methylation clocks for human aging have been proposed. Most of them are based on the Illumina 450K BeadChip platform, typically using the weighted average of tens or hundreds of CpG sites to predict chronological age. For instance, Hannum *et al.* developed a multivariate linear regression model using 71 CpG sites from blood DNA to predict biological age (Hannum *et al.* 2013). Horvath *et al.* proposed a universal DNA methylation clock, constructed using data from multiple tissues and cell types (Horvath 2013). This clock is widely used in various tissues and cell types for predicting biological age and serves as an important tool for aging and disease research. In addition to DNA methylation profiles, various other types of data were also incorporated into aging prediction model. For example, the Horvath2's Clock (also known as PhenoAge) combines clinical biochemical markers and DNA methylation data to predict biological age and assess individual health status and all-cause mortality risk (Levine *et al.* 2018). Levine's Clock (GrimAge) predicts biological age and mortality risk by combining multiple epigenetic markers, such as DNAm-based smoking pack-years and plasma protein markers, making it an important tool for evaluating health status and life expectancy (Lu *et al.* 2019). Other research aimed to predict ages for a specific population, e.g. the PedBE clock is a DNA methylation clock designed for children and adolescents, aiding in the study of early aging processes and health conditions (McEwen *et al.* 2020).

However, although widely used in clinical settings, the above array-based epigenetic clocks have several limitations. Firstly, DNA methylation array typically assesses methylation at predefined CpG sites, which constitute only a small subset of the total CpG sites in the genome scale (roughly 1.6% for the Illumina 450K array). The limited CpG site coverage would possibly miss important methylation changes associated with aging (Bell *et al.* 2019). Secondly, array-based techniques are actual semi-quantitative methods thus suffer from reduced detection sensitivity to low-abundant methylation changes, especially in regions with low CpG density or in heterogeneously methylated regions (Bibikova *et al.* 2009, Pidsley *et al.* 2013). Thirdly, array-based techniques show a limited dynamic range for detecting DNA methylation changes as compared with bisulfite sequencing techniques (Schumacher *et al.* 2006). Lastly, array-based clocks might not effectively capture the epigenetic heterogeneity within a sample, as they only take input methylation levels of independent and limited CpG sites.

Compared to methylation arrays, bisulfite sequencing (BS-seq) technology offers remarkable advantages, such as genome-wide CpG site coverage, single-base resolution, highly sensitive and unbiased quantification. However, to the best of our knowledge, no DNA methylation clock utilizing BS-seq data has been developed yet. In this study, we collected publicly available BS-seq data from multiple tissues and organs, and constructed an Elastic Net regression model to predict the methylation age of each sample. Based on the predicted methylation age, we explored the aging rate distribution across different disease states and their gender-dependence across different age groups. We also explored the cross-tissue prediction capability of the BS-clock by applying the model trained on one tissue type to other tissue types. These investigations enhance our understanding of the aging process and provide scientific evidence for personalized anti-aging intervention strategies.

## 2 Materials and methods

### 2.1 Data resources

We obtained DNA methylation sequencing data in mHap format for four human tissues (Blood, Brain, Lung, and Skin) from the DNA Methylation Haplotype Browser. Methylation levels and read coverage for each CpG site were calculated using the *beta* function from the mHapTools toolbox. The sample metadata, including age, gender, and disease state, were retrieved using the *getGEO* function based on the corresponding GEO accession numbers. In addition, we sourced RRBS data from 61 patients with cancer of unknown primary (CUP) from the GEO database (GSE233087) as an external validation dataset.

### 2.2 Hyperparameter selection

#### 2.2.1 Coverage threshold

We conducted a systematic analysis by varying the CpG site coverage threshold from 20 to 100 in increments of 5 to assess its impact on model performance. As the coverage threshold increased, the number of CpG sites decreased, leading to fluctuations in the correlation between predicted and chronological age (Supplementary Fig. S1D and E). Notably, the highest correlation was observed at coverage thresholds of 45 and 50. After careful consideration of both the number of CpG sites and the strength of the correlation, we concluded that a coverage threshold of 50 offered the optimal balance between feature selection and predictive accuracy.

#### 2.2.2 Correlation threshold

We first analyzed the overall distribution of correlations between mean methylation levels and chronological age, and found that most correlations were concentrated between −0.5 and 0.5 (Supplementary Fig. S1A). Based on this observation, we tested absolute correlation thresholds ranging from 0.1 to 0.5 in increments of 0.05 to investigate how the number of selected CpG sites and their predicted correlations with chronological age varied with different thresholds (Supplementary Fig. S1B and C). Our results demonstrated a general trend of decreasing CpG site as the correlation threshold increased. Based on this comprehensive analysis, we ultimately selected 0.2 as a balanced threshold. This choice ensures an adequate number of CpG sites while maintaining high predictive accuracy across all datasets.

### 2.3 Feature selection

For each tissue type, we retained CpG sites that were covered by >50 reads and had an absolute correlation >0.2 between mean methylation level and chronological age. Next, we employed the bootstrap method to select CpG sites. Initially, we performed 10-fold cross-validation on the entire dataset to determine the optimal hyperparameters for the Elastic Net model. Then, we bootstrapped the data by iteratively sampling with replacement from the original data and constructing an Elastic Net model using the previously determined optimal hyperparameters. We then calculated the frequency of each feature being selected in the 500 bootstrap models (i.e. the number of times the coefficients were nonzero). CpG sites selected in >50% of bootstrap models were chosen as final features.

### 2.4 The BS-clock model

Based on the CpG sites selected from the previous step, we established the following two Elastic Net regression models for each tissue type,

Model 1:

$$\hat{Y}=\beta_{0}+\sum_{i=1}^{p}\beta_{i}X_{i}+\sum_{j=1}^{q}\gamma_{j}D_{j}+\lambda_{1}\sum_{i=1}^{p}\left|\beta_{i}\right|+\lambda_{2}\sum_{i=1}^{p}\left|{\beta_{i}}^{2}\right|,$$

Model 2:

$$\hat{Y}=\beta_{0}+\sum_{i=1}^{p}\beta_{i}X_{i}+\lambda_{1}\sum_{i=1}^{p}\left|\beta_{i}\right|+\lambda_{2}\sum_{i=1}^{p}\left|{\beta_{i}}^{2}\right|.$$

In both models, $\hat{Y}$ represents the predicted methylation age, $X_{i}$ denotes the methylation level of the ith CpG site, $D_{j}$ is a binary variable indicating the occurrence of the jth disease type. $\lambda_{1}$ and $\lambda_{2}$ are regularization parameters for balancing feature selection and weight penalty effects. The only difference between two models is whether disease state $D_{j}$ is incorporated as a covariate. We used the *Leave-OneOut* function from sklearn.model_selection to predict the methylation age for each sample, treating each sample as the test set and the remaining samples as the training set. Within the training set, we used 5-fold cross-validation to determine the regularization parameters $\lambda_{1}$ and $\lambda_{2}\;$that minimized the mean squared error.

For Model 1 which includes disease state as a covariate, we evaluated the significance of the covariate by examining the coefficients from each LOOCV model and calculated the confidence interval for the covariate.

### 2.5 Performance evaluation

Two metrics were used to evaluate the model accuracy:

1. **Correlation with chronological age**
$$r=\mathrm{correlation}\left(\hat{Y},Y\right),$$
  where $\hat{Y}$ and Y are vectors of predicted and chronological ages across all samples.
2. **Mean absolute error (MAE)**
$$\mathrm{MAE}=\;\frac{1}{n}\sum_{i=1}^{n}\left|{\hat{Y}}_{i}-Y_{i}\right|,$$
  where ${\hat{Y}}_{i}$ and $Y_{i}$ are predicted and chronological for the ith sample, *n* is the number of samples.

### 2.6 Implementations of array-based methylation clocks

We implemented seven array-based DNA methylation clocks using the R package *methylclock_1.0.1*. DNAm age was estimated by specifying the ‘*clocks*’ parameter in the *DNAmAge* function as “*Horvath*”, “*Hannum*”, “*Levine*”, “*SkinHorvath*”, “*PedBE*”, “*Wu*”, and “*TL*”, respectively. Due to the limited CpG coverage in array-based clocks, *min.prec* parameter, which specifies the minimum coincidence percentage required to perform the calculation, was set to 0.2.

### 2.7 Gene functional enrichment analysis

#### 2.7.1 GO enrichment analysis

Spearman correlation between methylation level of each CpG site and chronological age was calculated, retaining only CpG sites with an absolute correlation >0.2. Gene annotation was performed using the *TxDb.Hsapiens.UCSC.hg19.knownGene* database and *annotatePeak* function from ChIPseeker_1.41.0. GO enrichment analysis on the gene list with adjusted *P*-values <0.05 was performed using the *org*. *Hs.eg.db* database, visualized with ggplot2_3.5.0.

#### 2.7.2 GREAT functional enrichment analysis

We used the Genomic Regions Enrichment of Annotations Tool (GREAT) to analyze age-related CpG sites. Specifically, the top 500, 1000, 2000, and 3000 CpG sites showing the highest positively and negatively correlation with age were calculated based on all samples. GREAT utilizes a hypergeometric test to assess the functional annotations of these sites, using the genomic regions of these CpG sites as the test regions and the entire genome as the background. Gene set enrichment analysis for GO Biological Process, Cellular Component, Molecular Function, and Human Phenotype were performed. Significantly enriched pathways appearing in both normal and disease samples were retained, visualized using the ComplexHeatmap_2.16.0 package in R.

### 2.8 Protein–protein interaction analysis

Protein–protein interaction (PPI) analysis for Blood, Brain, Lung, and Skin tissues was conducted. Gene annotation results from GO enrichment analysis were filtered, retaining genes with a Spearman correlation ≥0.45 and a *P*-value <1e−3 for blood, and a *P*-value <1e−2 for lung. Gene symbols were merged into a single list. A STRINGdb object was initialized with specified parameters (version 11.5, human species, score threshold of 400) and mapped gene symbols to STRINGdb protein IDs. Incomplete cases were filtered, and protein interaction data from STRINGdb was retrieved.

Interactions, including protein pairs, interaction scores, and tissue sources, were saved as a CSV file. The data was visualized using Cytoscape_v3.10.2 software, displaying PPI networks specific to each tissue type.

## 3 Results

### 3.1 Overview of BS-clock model and prediction accuracy

We collected a total of 529 BS-seq samples from the DNA Methylation Haplotype Browser (Hong *et al.* 2024), encompassing normal and various disease states from blood (*N* = 194), brain (*N* = 253), lung (*N* = 63), and skin (*N* = 19) tissues. One major challenge in handling BS-seq data in large cohort studies is the substantial file size, with a typical whole genome bisulfite sequencing (WGBS) sample producing approximately 10 GB of raw reads data at sufficient sequencing coverage. To address this issue, we stored the BS-seq data in mHap, a highly compressed file format for DNA methylation BS-seq data developed by our group (Zhang *et al.* 2021). Unlike the BAM format that stores raw sequencing reads, the mHap format preserves methylation status of sequencing reads as aggregated binary strings, achieving up to 140-fold compression compared to the original bam file while retaining complete read-level CpG methylation information. We also developed the mHapTK toolkit for manipulating mHap data (Ding *et al.* 2022), including functions for extracting mean methylation of each CpG site, calculating haplotype statistics, and visualizing data.

**Figure 1.**
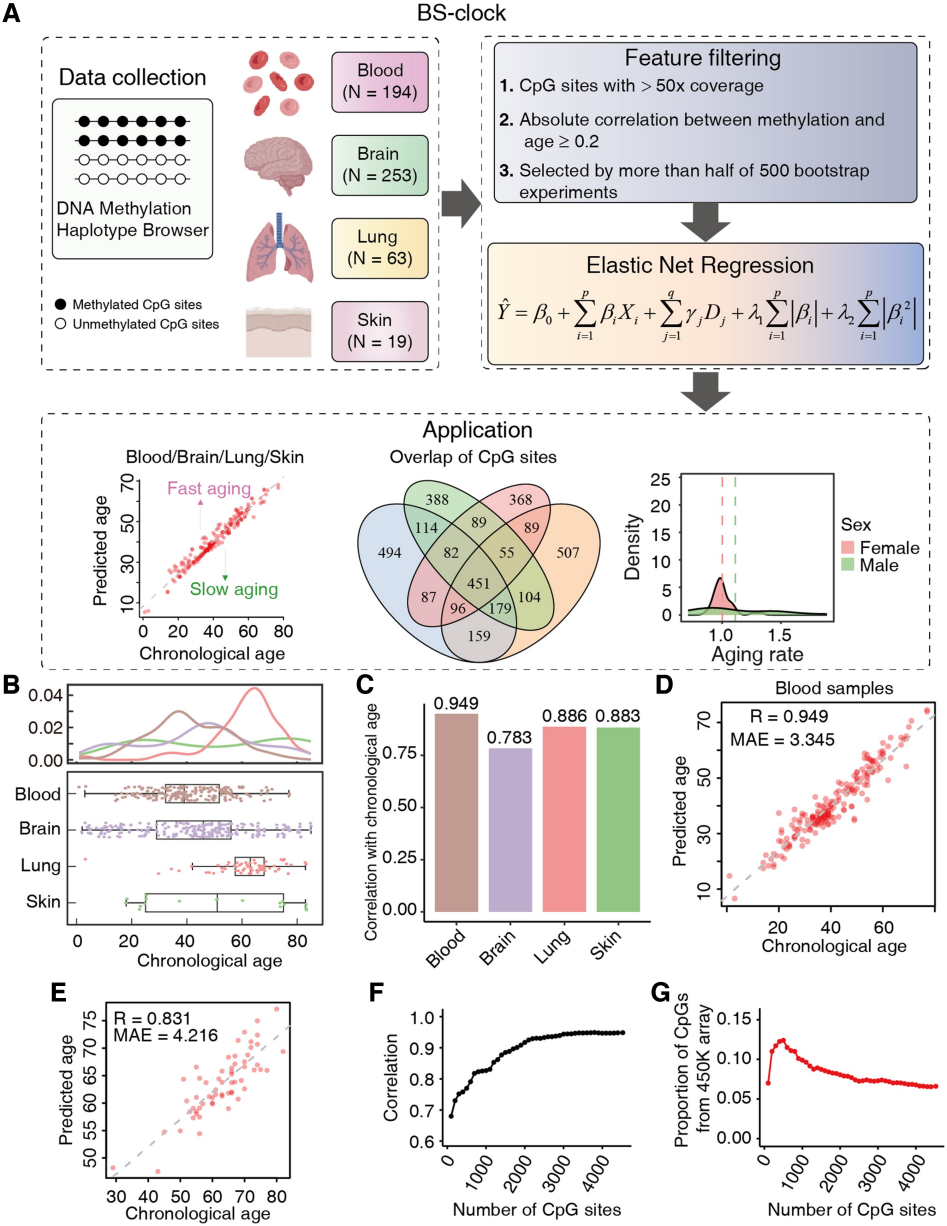
Overview of the BS-clock workflow and aging prediction performance in multiple tissues. (A) Workflow of the BS-clock pipeline. DNA methylation BS-seq data from 529 samples in the mHap format were retrieved from the DNA Methylation Haplotype Browser. DNA methylation profiles at the CpG site level were extracted and filtered by stringent criteria, including read coverage, correlation with age, and occurrence in multiple bootstrap models. The selected features were then input into an Elastic Net regression model, with disease status treated as covariates. The trained BS-clock model can be used to calculate the aging rate of individual samples and investigate differences in aging across various populations. (B) Chronological age distribution of samples from four tissue types. (C) Correlations between chronological and predicted ages in four tissues. (D) Scatter plot demonstrating the alignment between predicted ages and chronological ages in blood samples. (E) Scatter plot of BS-clock's predicted ages and chronological ages on an independent test set. (F, G) Correlation (F) and the proportion of CpG sites included in the 450K array (G) (*y*-axis) are plotted against the number of CpG sites in blood samples.

We developed genome-wide epigenetic clocks (BS-clock) for these tissues (Fig. 1A). For each tissue type, we selected CpG sites with at least 50-fold coverage reads in each sample and a mean methylation correlation over 0.2 with chronological age (Supplementary Fig. S1A–E). Using a resampling method 500 times, we retained CpG sites selected by more than half of the bootstrap experiments. The selected CpG sites are input to an Elastic Net regression model, and we used leave-one-out cross-validation (LOOCV) to estimate the performance of our model. The BS-clocks not only predicted methylation age across tissues but also identified tissue-specific CpG sites and their functions. We examined the differences in aging rates (defined as the ratio of predicted age to chronological age) across genders and health statuses, and explored the overlap of selected CpG sites across different tissue models.

We detailed the age distribution of samples from four tissue types (Fig. 1B). Ages ranged from 1 to 85 years, with lung samples predominantly between 40 and 80 years. Regarding disease status, most blood samples were either normal (63) or leukemia (104), while brain samples were primary from normal (121) or schizophrenia (66) (Supplementary Fig. S1F). Lung and skin samples were relatively small and consisted of cancer and normal tissues, respectively. To assess the impact of disease states on our model, we trained two models by excluding or including disease states as covariates in the Elastic Net model, while keeping other features and hyperparameters fixed. By including disease states as features, our BS-clock model accurately predicted chronological age, achieving correlations of 0.949 for blood, 0.783 for brain, 0.886 for lung, and 0.883 for skin samples (Fig. 1C, Supplementary Fig. S1G–I), which were approximately 5% higher than models without disease information as input (Supplementary Fig. S1J–N). Thus, we refer to BS-clock as covariate model unless otherwise specified. The mean absolute error (MAE) of BS-clock was 3.345 years for blood samples (Fig. 1D), and 10.861, 3.784, and 10.87 years for other three tissue types (Supplementary Fig. S1G–I), demonstrating the high accuracy and robustness across tissues. To further validate our model using external validation data, we downloaded RRBS data of 61 blood samples from patients with carcinoma of unknown primary (CUP) from the GEO database. Our BS-clock still achieved a high prediction accuracy of 0.831 and an MAE of 4.216 (Fig. 1E) on this independent dataset.

We next sought to explore whether a small set of CpG sites could yield reliable age prediction. Using blood samples as an example, we plotted prediction correlations with chronological age as the number of CpG sites increased, where CpG sites are ranked by their correlations with age. The analysis revealed that just a few hundreds of CpG sites could achieve relatively high accuracy (Correlation = 0.679, Fig. 1F), while the accuracy stabilized with >2000 CpG sites (Correlation > 0.9). Notably, only a small proportion of CpG sites (<10%) came from the 450K array (Fig. 1G), highlighting the significance of developing BS-seq based aging clocks. We also employed a model-based feature selection method, LASSO, to identify a subset of CpG sites that have the most predictive value to the target variable. This approach effectively identified a more reduced set of 277 CpG sites, achieving an age prediction accuracy of 0.934 using the same Elastic Net regression model (Supplementary Fig. S1O and P).

### 3.2 Comparison of BS-clock with array-based methylation clocks

**Figure 2.**
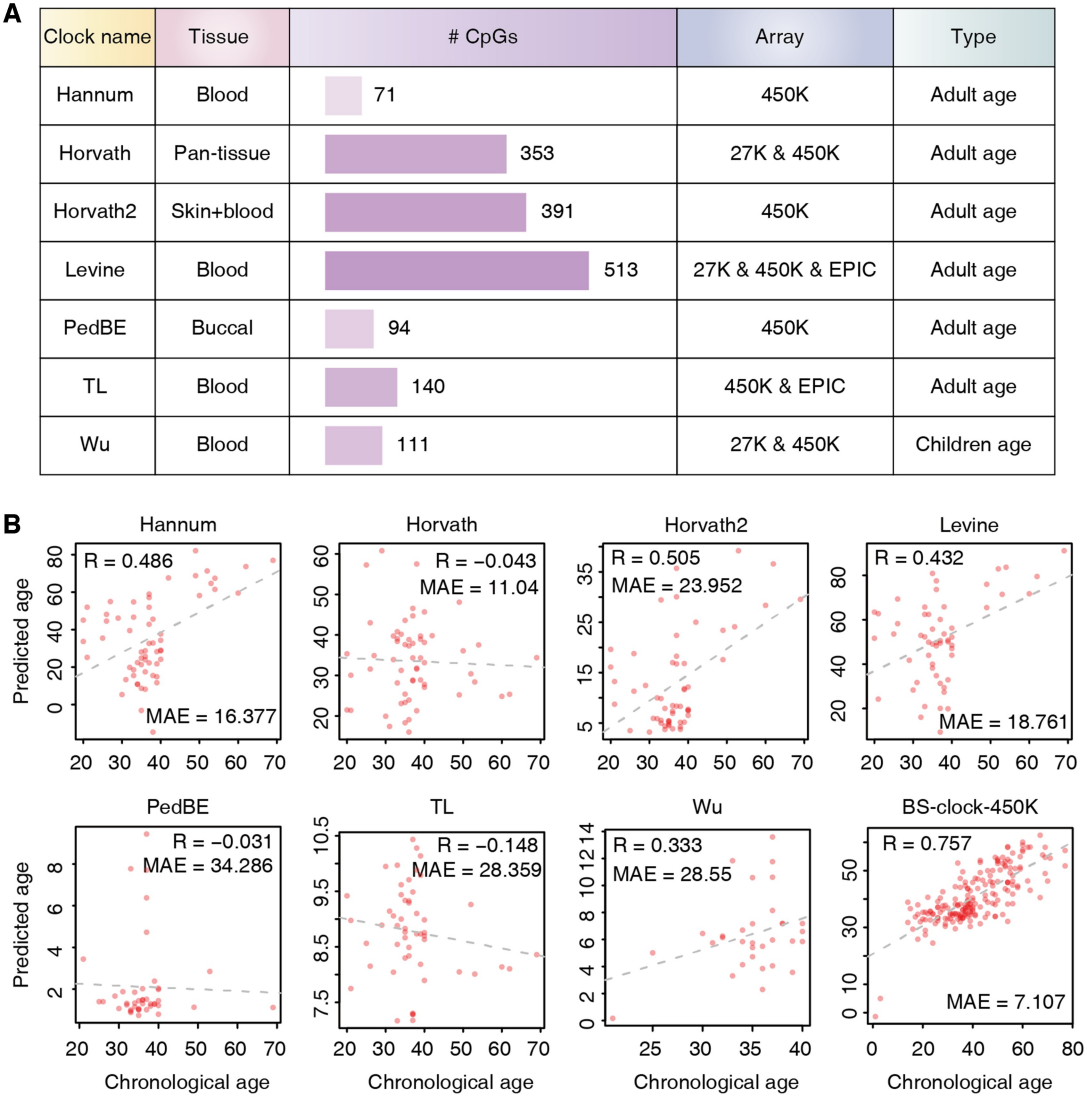
Comparison of various DNA methylation clocks and their predictive performance on BS-seq data. (A) Summary of array-based DNA methylation clocks, including target tissue types, the numbers of CpG site features utilized, array types, and the population each clock is designed for. (B) Performance of DNA methylation clocks on blood sample based on BS-seq data. To facilitate the application of array-based methylation clocks, CpG sites in BS-seq were restricted to those present in 450K array. For equitable comparison, BS-clock was also confined to CpG sites within 450K array.

As currently no epigenetic clock designed specifically for BS-seq data, we compared our model with existing array-based epigenetic clocks, including Hannum’s clock, Horvath’s clock, Horvath2’s clock, Levine’s clock, PedBE clock, TL’s clock, and Wu’s clock (Fig. 2A). These clocks target different tissue types, use diverse array platforms (27K, 450K, or EPIC array), and rely on specific CpG sites (ranging from 71 to 513). Note that although BS-seq offers potential genome-wide coverage, it does not guarantee sufficient coverage for each single CpG site used in array-based clocks. To ensure robust and reliable estimates, we input CpG sites with at least 5-fold coverage in BS-seq into these array-based clocks. For the BS-clock designed for BS-seq data, we restricted the input to CpG sites probed in the 450K array, and termed this reduced version BS-clock-450K.

We found that array-based clocks exhibited varying and relatively low prediction performance on BS-seq data (Fig. 2B). For example, Horvath’s clock, PedBE clock, and TL’s clock showed correlations of −0.043, −0.031, and −0.148 to chronological age, respectively. Although Hannum’s clock, Horvath2’s clock, Levine’s clock, and Wu’s clock achieved higher correlations, the resulting MAEs are very high, indicating biased prediction toward to a few highly affected samples. The suboptimal performance of array-based clocks on BS-seq data may arise from differences in CpG site coverage, as BS-seq may not capture the same CpG sites as array-based methods. Meanwhile, batch effects, technical variations in experimental protocols, and data processing methods can also introduce inconsistencies (Leek *et al.* 2010, Qin *et al.* 2016), further affecting the accuracy of age predictions.

In contrast, BS-clock performed remain better on 450K array-probed CpG sites, with a correlation of 0.757 and a lower MAE of 7.107 (Fig. 2B). The result is much lower than that by the full version of BS-clock, underscoring the importance of whole genome-scale BS-seq data for accurate aging prediction. In summary, existing array-based methylation clocks cannot be directly applied to BS-seq data, and our BS-clock still achieved reliable methylation age prediction by using a subset of CpG sites from 450K array.

### 3.3 DNA methylation aging rate and its clinical associations

**Figure 3.**
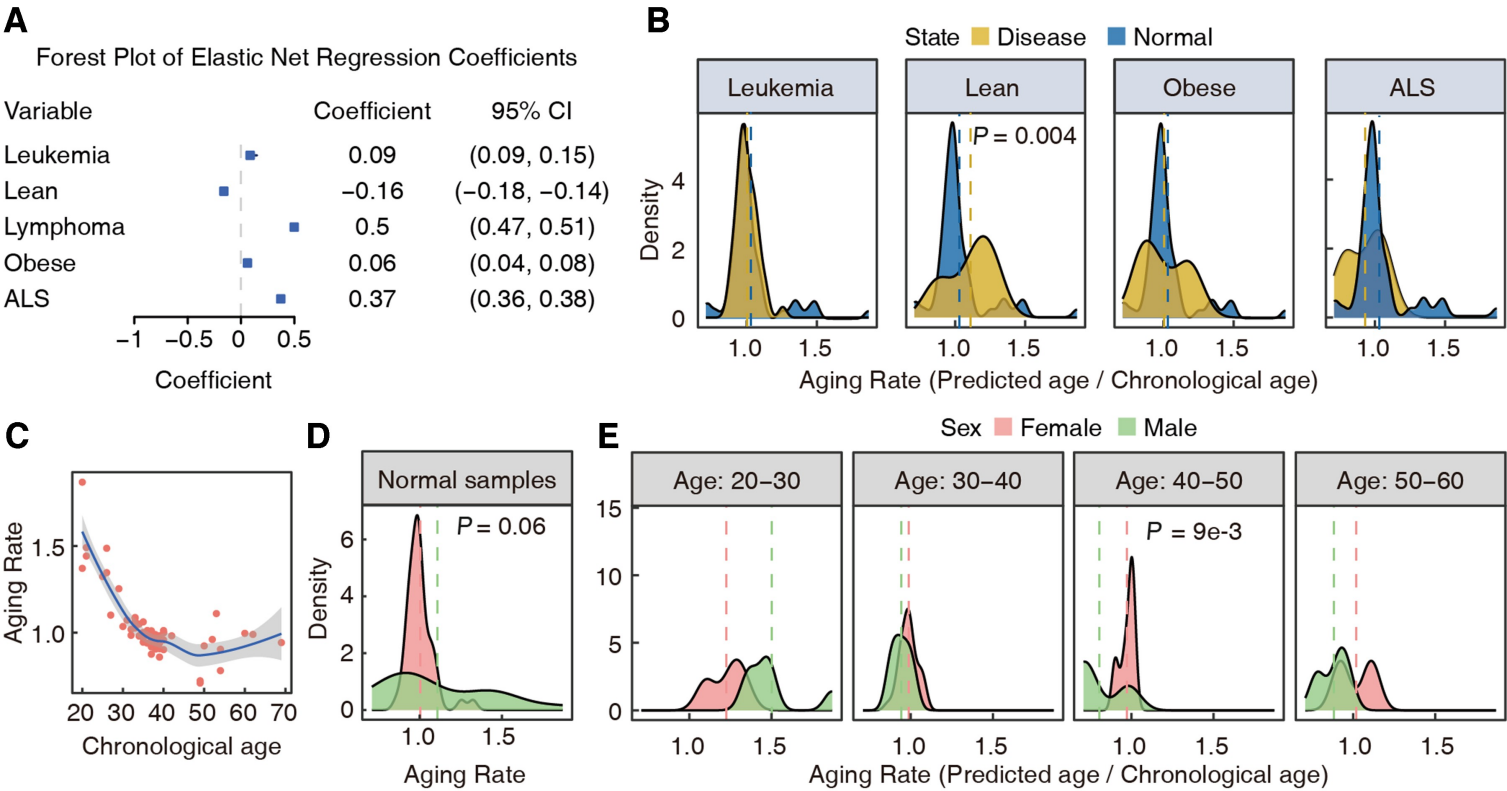
Associations between clinical phenotypes and aging. (A) Coefficients and 95% confident intervals of disease status terms in the Elastic Net regression model. (B) Comparison of Aging rates, defined as the ratio between predicted age and chronological age, across various disease statuses and healthy samples. ALS, Amyotrophic Lateral Sclerosis. (C) Aging rate trend with the increase of chronological age. (D) Differences in aging rates between males and females in normal samples. (E) Differences in aging rates between males and females at different age ranges.

The BS-clock incorporated disease states as covariates in the Elastic Net regression model, enabling us to investigate the impacts of these factor on aging by examining their coefficients in the regression model. According to the forest plot, all five disease states show significant effects on aging (Fig. 3A). Among them, lymphoma and ALS (Amyotrophic Lateral Sclerosis) are associated with accelerated aging. Leukemia and obese also show significant but weaker effects, while leanness indicates deceleration of aging. This is expected since lymphoma is often associate with clonal restriction in hematopoietic stem cell, which could promote age-related defects including cancer (Sarkozy *et al.* 2015). Meanwhile, ALS, a devastating neurodegenerative disorder, includes aging as a major risk factor. Recent studies indicated that ALS, characterized by the progressive loss of motor neurons, leading to muscle weakness and atrophy (Ciervo *et al.* 2017), is influenced by age-related molecular changes such as dysregulation of RNA metabolism and increased oxidative stress (Konovalova *et al.* 2019, Appel *et al.* 2021, Zuo *et al.* 2021). On the contrary, maintaining lean body mass is well-documented to beneficial aging process, associated with better physical function and metabolic health of human body, including improved insulin sensitivity, better lipid profiles and lower inflammation levels. The above conclusions were also confirmed by comparing the aging rates (calculated as the ratio between predicted and chronological ages) between health and different disease groups (Fig. 3B).

We next sought to examine the influence of age and gender on aging rate inferred from blood samples, defined as the ratio of predicted to chronological age. Rates above 1 indicate accelerated aging, while rates below 1 suggest slower aging. We first examined the influence of age and disease on aging rates of male and female samples. To this aim, we calculated the aging rate for each sample and plotted them against chronological ages. A high average aging rate (over 1.5) was observed at age 20, which is consistent with previous report that adolescents show accelerate aging (Hoare *et al.* 2022). The average aging rate showed a sharp decrease from 20 to 30 and stabilized around 1.0 after 30. Two distinct declines in the aging rate were observed between ages of 20–30 and 40–50 (Fig. 3C). Gender was also an important factor influencing aging rate, which was discovered independently by different groups (Lunenfeld 2001). However, a weak difference (*P *=* *0.06) was found in aging rate between two sex groups probably due to the limited sample size (Fig. 3D). When samples were divided into ten-year intervals, interesting age-dependent patterns emerged. There was no significant difference in the age range 30–40, but in the range 40–50, females aged faster than males (*P *=* *9e−3). In age range 50–60, female aging rates showed a bimodal distribution, with some females aging faster or slower than the general population (Fig. 3E).

The impact of disease states on aging rates was also examined (Supplementary Fig. S2A). Interestingly, in conditions like leanness and obesity, females aged faster than males (Supplementary Fig. S2B). This suggests that females should be more mindful of diet, avoiding extremes that can burden the body and affect lifespan. In healthy individuals, males aged faster, consistent with previous studies (Hägg and Jylhävä 2021). The results on ‘age discrepancy’, defined as the difference between predicted age and chronological age, are shown in Supplementary Fig. S3A and B. Results for models without disease states as covariate are in Supplementary Fig. S4A and B. These analyses indicated that aging rates across age stages and diseases helps identify factors that accelerate or decelerate aging, aiding in developing personalized anti-aging strategies to enhance health span and quality of life.

### 3.4 Functional enrichment of selected CpG sites by BS-clock

Age-related CpG sites are crucial for understanding aging mechanisms. In our analysis of blood samples, we identified 176 157 CpG sites that exhibited >50-fold read coverage and the absolute value of Spearman correlation with age exceeds 0.2. From these, BS-clock selected 4527 sites as having nonzero coefficients in at least half of 500 bootstrap experiments. To better characterize the genomic context of these selected CpG sites, we conducted a detailed analysis of the genomic region and genic annotations of them across various tissue samples (Supplementary Fig. S5). Our findings revealed that a significant proportion of age-related CpG sites in blood, brain, and skin tissues were annotated as located in InterCGI and Intergenic regions. Notably, blood samples showed a high concentration of CpG sites in InterCGI, CpG Islands, CpG Shores, and gene functional regions including Genes 1–5 kb, Exons, Intergenic and Introns. This distribution indicates that the selected CpG sites are not only genome-wide but also enriched in regions associated with gene regulation and function, which reinforces the validity of our model’s design.

**Figure 4.**
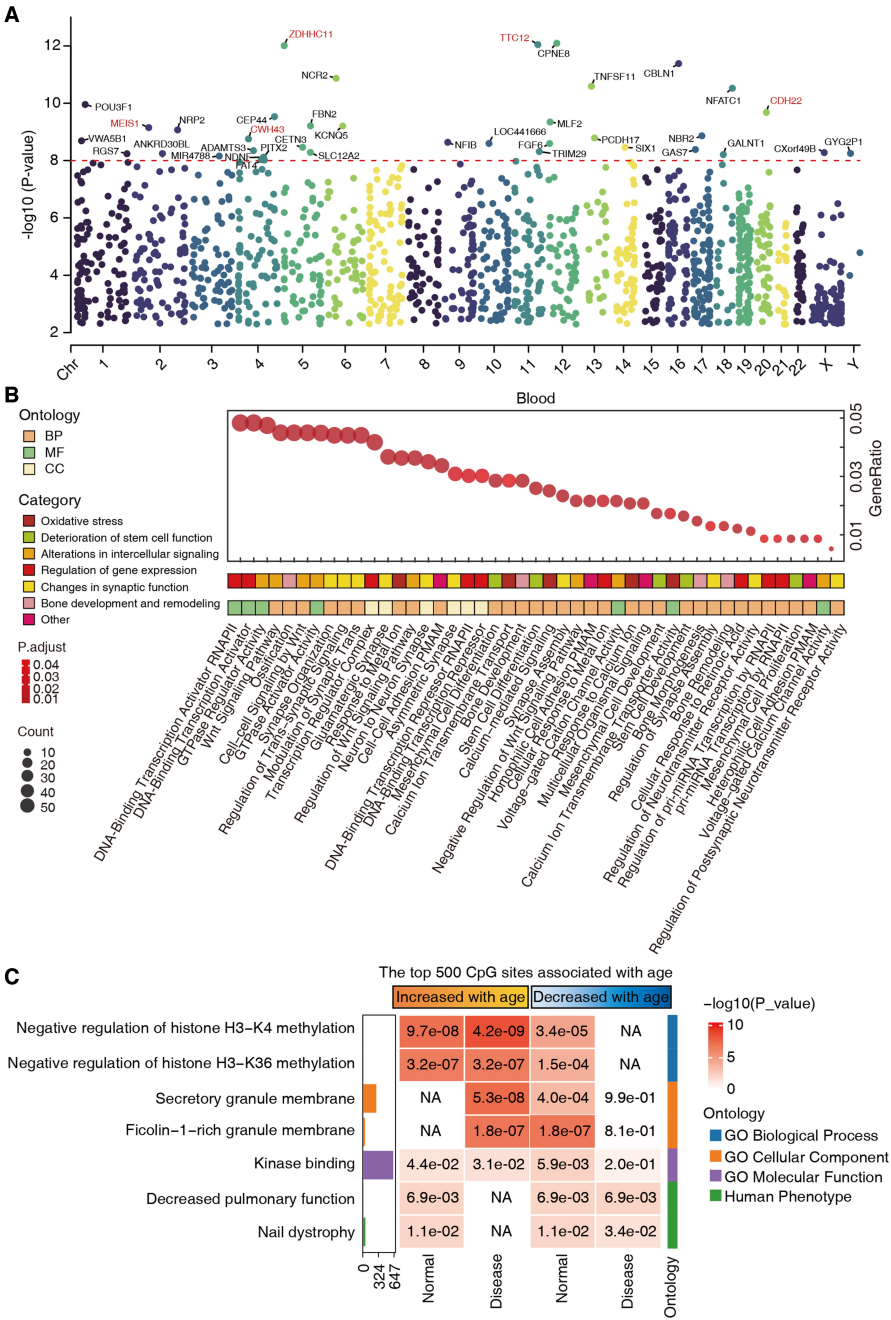
Functional enrichment analysis of the selected CpG site features. (A) Manhattan plot displaying *P*-values of selected genes by BS-clock using a correlation test, with genes showing significant *P*-values below 1e−8 highlighted. (B) Gene Ontology (GO) enrichment analysis of genes with *P*-values below 0.05. (C) GREAT functional enrichment analysis was based on the top 500 CpGs that increased or decreased with age from normal and disease samples. The *y*-axis lists the name of a functional gene set/biological pathway, sorted by ontology and the most significant *P*-value within each ontology. The bar plots in the first column report the total number of genes at each studied gene set. The heatmap color codes −log10 (*P*-value).

Next, we illustrate the final selected genes in a Manhattan plot (Fig. 4A), highlighting those with significant *P*-values below 1e−8. Several top-ranking genes, such as MEIS1, ZDHHC11, CWH43, TTC12, and CDH22, have been supported by recent studies as playing roles in various cancers and aging-related processes. For instance, MEIS1 is essential for sustaining the self-renewal and differentiation capacity of hematopoietic stem cells, which is critical for tissue regeneration and homeostasis during aging (Yao *et al.* 2021). In addition, abnormal expression of MEIS1 has been linked to various cancers and neurodegenerative diseases, such as restless legs syndrome (Sarayloo *et al.* 2019, Leu *et al.* 2024). Results for brain, lung, and skin samples are shown in Supplementary Fig. S6.

Gene Ontology (GO) enrichment analysis of genes with *P*-values below 0.05 revealed critical aging-related functions and pathways, such as oxidative stress, stem cell function decline, altered cell signaling, and gene expression regulation (Fig. 4B). Key processes include metal ion response, calcium signaling, stem cell development, Wnt signaling, and gene expression regulation. These insights enhance understanding of aging and identify potential anti-aging intervention targets. Furthermore, we used the Genomic Regions Enrichment of Annotations Tools (GREAT) to annotate the potential function of age-related sites in normal and disease samples. We sought to identify biological processes and pathways potentially associated with the top 500 positively and negatively age-related CpG sites. Results showed significant enrichment in functions like histone methylation regulation, kinase binding, and pulmonary function, emphasizing their importance in normal and disease states (Fig. 4C). The results of the top 1000, 2000, and 3000 CpG sites that were positively and negatively correlated with age are shown in Supplementary Fig. S7.

### 3.5 Cross-tissue aging rate prediction by BS-clock

**Figure 5.**
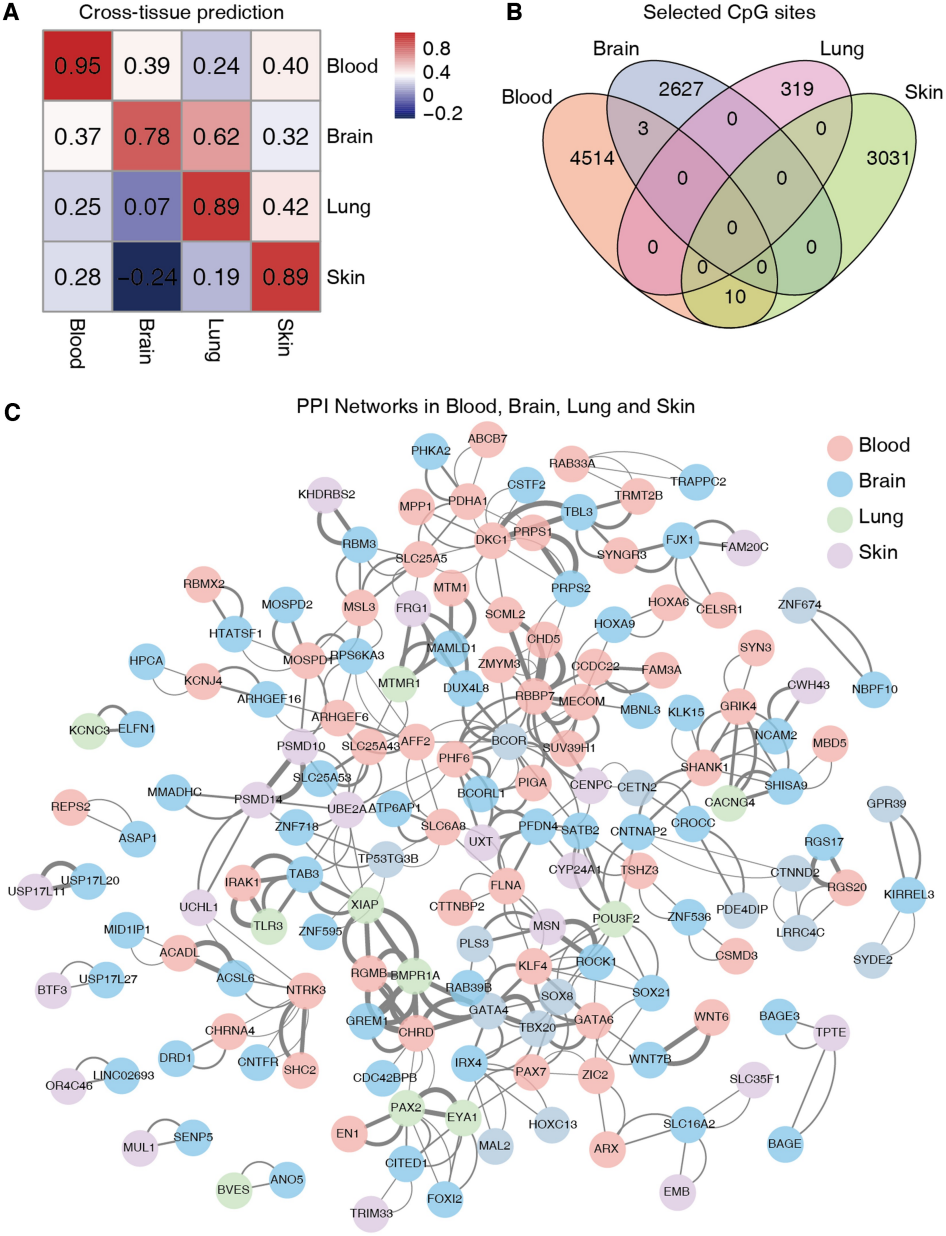
Performance of BS-clock across tissue types. (A) Cross-tissue prediction accuracies of BS-clocks trained from blood, brain, lung, and skin samples. (B) Overlap of selected CpG sites by BS-clocks trained from four tissue types. (C) Protein–protein interaction network of the corresponding genes adopted by four BS-clocks.

We next investigated the capability of BS-clock for cross-tissue prediction, specifically whether the BS-clock trained from easily accessible blood samples could be extended to other tissues. To this aim, we tested the model trained on each individual tissue type to other tissues from blood, brain, lung, and skin samples (Fig. 5A). An overall weak (or even negative) correlation was observed for cross-tissue prediction compared to within-tissue prediction, indicating the heterogeneity of aging mechanisms across different tissues. For example, the BS-clock trained from blood samples achieved correlations of 0.39, 0.24, and 0.40 for brain, lung and skin samples, respectively (Fig. 5A). A deeper analysis of the CpG site features used in four BS-clock models revealed that, although each model employed thousands of CpG sites (blood having 4514 unique sites, brain 2627, lung 319, and skin 3031), their pairwise overlaps were low (Fig. 5B). The lack of common CpG sites may explain the poor generalization of the blood-trained model.

GO enrichment analysis of tissue-specific CpG sites revealed significant functional differences. In skin, enriched functions included protease activity, suggesting relevance to protein metabolism and repair. Blood samples showed enrichment in mitochondrial function, GTPase activity, transcription factor binding, and synaptic membrane, indicating roles in energy metabolism, signaling, and gene regulation. Lung samples were enriched in axon development, synaptic transmission, ossification, and transcription regulation, highlighting roles in neural and bone processes (Supplementary Fig. S8A–C). However, we did not find significant functional enrichment in brain samples, probably due to the limited brain-specific CpG sites or their dispersed distribution across various functional pathways. To further investigate it, we loosened the threshold of the enrichGO function by increasing the q-value cutoff from 0.05 to 0.2, and found enriched pathways related to cell−cell adhesion mediator activity, cysteine-type endopeptidase activity, and thiol-dependent ubiquitin-specific protease activity. These pathways play crucial roles in modulating aging processes, influencing cellular interactions, proteostasis, and the regulation of protein turnover (Supplementary Fig. S8D).

Protein–protein interaction (PPI) network analysis of highly age-correlated CpG sites revealed significant gene interactions across tissues (Fig. 5C). Key interacting genes included RGMB, CHRD, GATA6, GREM1, BMPR1A, XIAP, MTM1, MAMLD1, DUX4L8, MTMR1, FRG1, SCML2, CHD5, RBBP7, and MECOM, involved in growth, development, and extracellular matrix regulation (Qian *et al.* 1993, Laporte *et al.* 1996, Morrisey *et al.* 1998, Bachiller *et al.* 2000, Mishina *et al.* 2002, Michos *et al.* 2004, Babitt *et al.* 2005, Bagchi *et al.* 2007, Liang and Wang 2020). These insights aid in understanding aging gene interactions and identifying anti-aging targets.

## 4 Discussion

The development of an accurate and reliable aging clock significantly enhances our understanding of the human aging process and has profound implications for both research and clinical applications. By providing a more precise measure of an individual’s biological age, an aging clock can inform us the potential age-related risks and early warnings for age-related diseases. In this study, we developed an accurate epigenetic aging clock, BS-clock, using high coverage bisulfite sequencing data. Unlike array-based methods which are limited to pre-defined probe sets, BS-seq can potentially capture all CpG sites in the genome, thus allowing for the discovery of novel age-related methylation changes that might be missed by arrays. Meanwhile, sequencing-based approaches can be directly applied to other model organisms or even nonmodel organisms, as they do not require species-specific array designs. This advantage broadens the potential applications of aging clocks across different biological systems. Moreover, sequencing data can be readily integrated with other types of genomics data obtained through sequencing, such as genetic variation, gene expression, and alternative splicing, facilitating more comprehensive multi-omics analyses of aging.

Based on the BS-clock models trained from four important tissue types, our analyses identified factors that have substantial influence on human aging and highlighted differences in aging rates across various age groups and disease states. This reveals gender-specific aging rate differences in specific age groups, offering a basis for personalized health management and anti-aging interventions. By analyzing aging rate differences across various disease states, it underscores the importance of a healthy lifestyle and disease prevention, particularly emphasizing dietary health in women. Furthermore, we comprehensively evaluated the performance of BS-clock in cross-tissue prediction. When models trained on one tissue type were tested on other tissues, we observed poor cross-tissue predictive generalizability, likely due to variations in CpG sites among tissues. The validation of the BS-clock model's predictive performance across different tissue samples sets a reference for developing more generalizable aging prediction models. Functional enrichment and PPI network analysis identified key aging-related genes and their interactions, providing crucial insights into the mechanisms of aging. Based on these findings, we can explore the application of aging rate predictions in personalized health management and anti-aging interventions to support improved health span and quality of life.

However, this study suffers from several limitations. First, the sample primarily consists of blood samples, with relatively fewer samples from other tissues, potentially affecting the generalizability of the results. Future research could expand in the following directions: collecting more samples from various tissues and age groups to enhance model generalizability and predictive accuracy, incorporating advanced machine learning algorithms and model optimization techniques to improve performance, especially in cross-tissue predictions, and further exploring the function of CpG sites in different tissues to identify more aging-related key genes and their mechanisms. Second, high-coverage sequencing-based approaches (especially whole-genome bisulfite sequencing) have the drawbacks of higher cost and more complicate data analysis. It is more practical to first identify age-related DNA methylation sites and regions through sequencing-based aging models such as BS-clock, and then design a targeted array to reduce the sequencing cost. Third, in addition to mean methylation for each CpG site used in our model, read-level methylation metrics (also known as methylation haplotype statistics), such as proportion of discordant reads (PDR), Cell Heterogeneity-Adjusted cLonal Methylation (CHALM) (Xu *et al.* 2021), methylation concurrence ratio (MCR) (Shi *et al.* 2021) and Methylation Haplotype Load (MHL) (Landau *et al.* 2014), has demonstrated superior capabilities in capturing the inherent heterogeneity of complex tissues. Leveraging these advanced metrics would potentially offer a more comprehensive and accurate representation of epigenetic changes associated with aging. Developing models to incorporate these age-related features would be the future direction of our work. Fourth, our BS-clock model employed the typical Elastic Net regression, which was widely used in aging prediction based on array data. It has the advantages of interpretability, computational efficiency, and facilitating feature selection through regularization, but fails to capture the intricate nonlinear relationships in data. More advanced machine-learning models, such as Random Forests, Gradient Boosting, Deep Neural Network, or ensemble-based methods, should be further employed into the aging prediction model to address these limitations.

## Supplementary Material

btae656_Supplementary_Data
