# Supplementary material for: BS-clock, advancing epigenetic age prediction with high-resolution DNA methylation bisulfite sequencing data
Source: Bioinformatics. 2024 Nov 5;40(11):btae656. doi: 10.1093/bioinformatics/btae656 (PMC11572488; doi:10.1093/bioinformatics/btae656)
Supplement: btae656_Supplementary_Data [file btae656_supplementary_data.zip › supplementary material.docx]

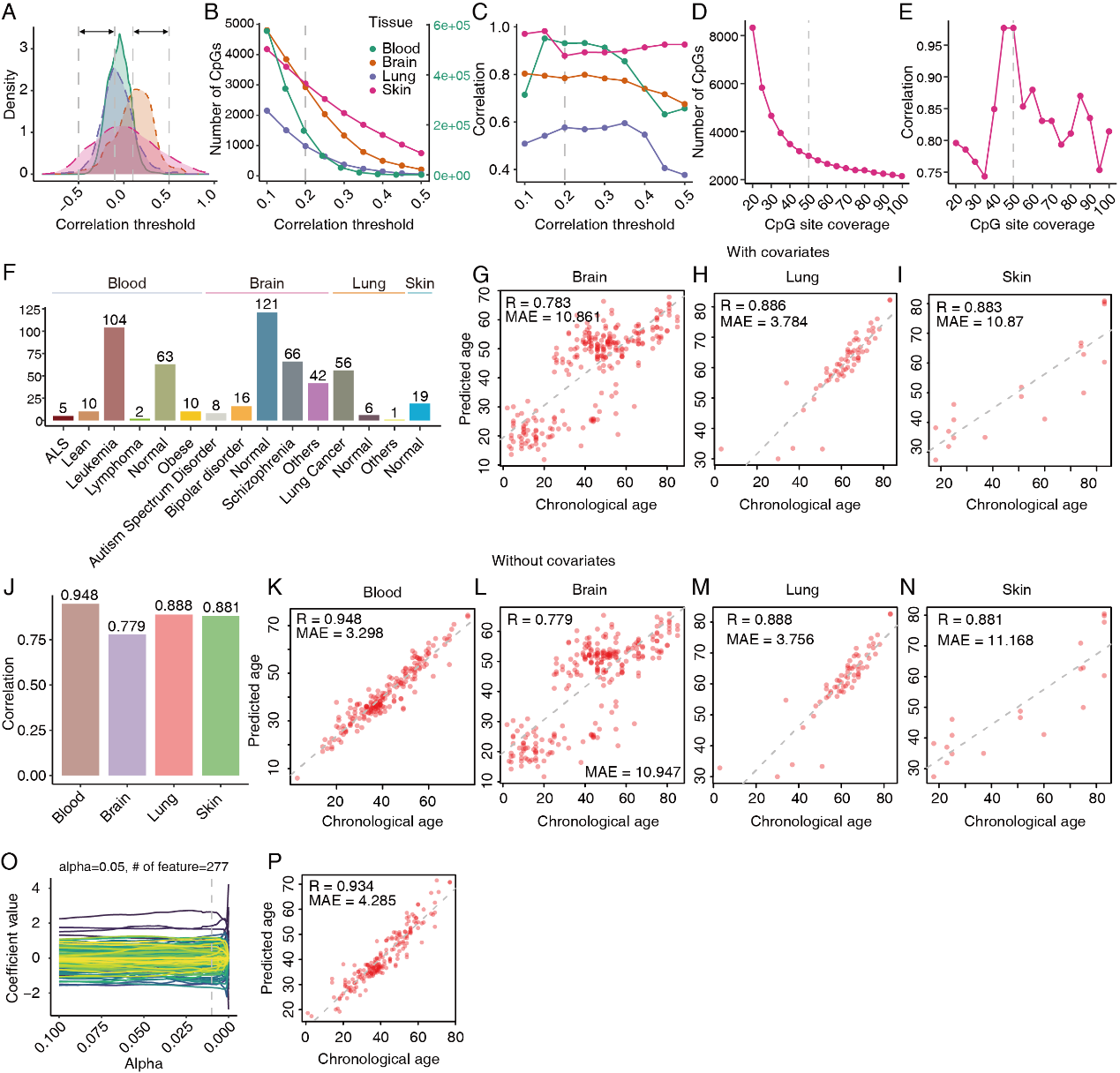


**Supplementary Figure 1. Determination of hyperparameters and performance of BS-clock in predicting aging across multiple tissues with and without covariates.** (A) Density plots of correlation distributions across four tissues, with grey dashed lines indicating correlation thresholds of ±0.2 and ±0.5. (B-C) The number of CpGs (B) and the correlation between chronological age and predicted age (C) as a function of the correlation threshold (ranging from 0.1 to 0.5 in increments of 0.05). The grey dashed line indicates a correlation threshold of 0.2. (D-E) The number of CpGs (D) and the correlation between chronological age and predicted age (E) as a function of the CpG site coverage (ranging from 20 to 100 in increments of 5). The grey dashed line indicates a CpG site coverage of 50. (F) Number of samples in different disease states across four tissue types. Others refer to samples from disease types that are not closely related to aging. (G-I) Scatter plots of the correlation between age predicted by the model with covariates and chronological age for brain (G), lung (H), and skin (I) tissues. (J) Correlations between chronological and predicted ages in four tissues. (K-N) Scatter plots depicting the correlation between age predicted by the model without covariates and chronological age for blood (K), brain (L), lung (M), and skin (N) tissues. (O) Coefficients of the Lasso model as a function of alpha values (0 to 0.1). A vertical dashed gray line at alpha = 0.01 indicates the selected value, along with the number of features retained. (P) Scatter plot of predicted age and chronological age using features selected by Lasso for the BS-clock model.


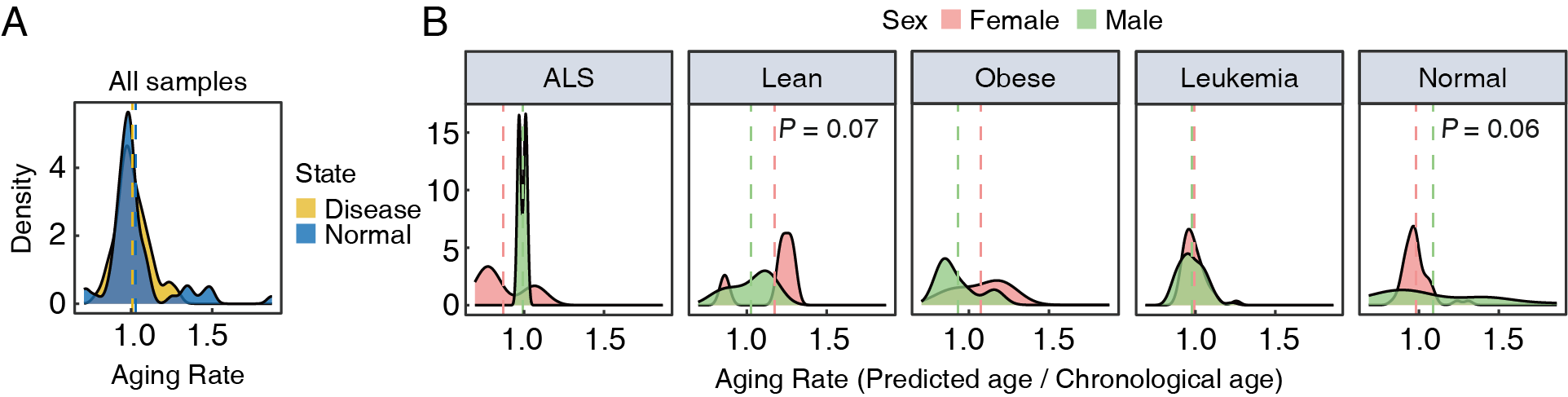


**Supplementary Figure 2. Overview of aging rates in different disease states.** (A) Differences in aging rates between normal and diseased blood samples. (B) Differences in aging rates between males and females across various disease states.


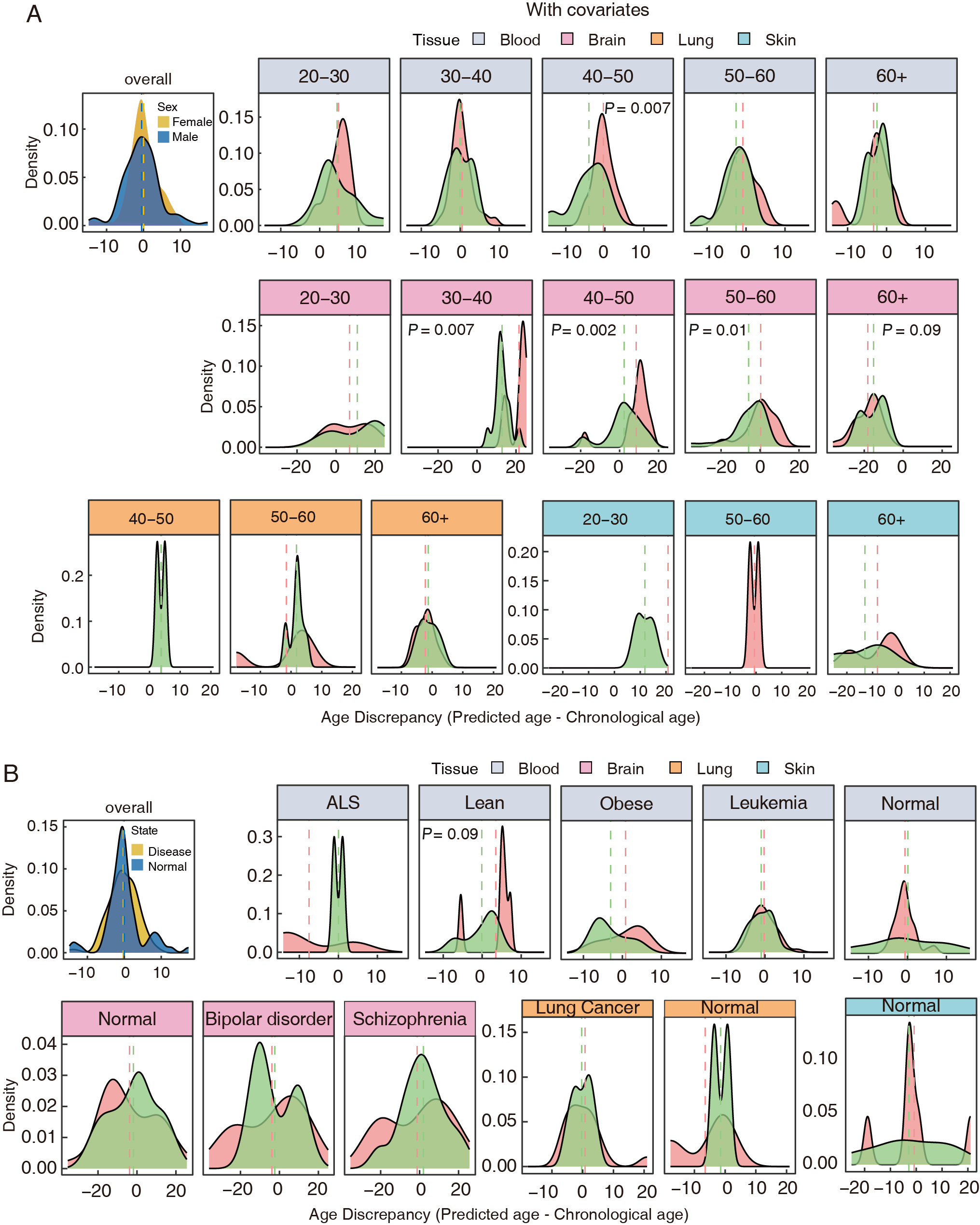


**Supplementary Figure 3. Associations between clinical phenotypes and aging using a model with disease status as a covariate.**  (A) Differences in age discrepancy between males and females at different age ranges in four tissues. (B) Differences in age discrepancy between males and females under different disease states in four tissues. The age discrepancy is defined as the difference between the predicted age and the chronological age.


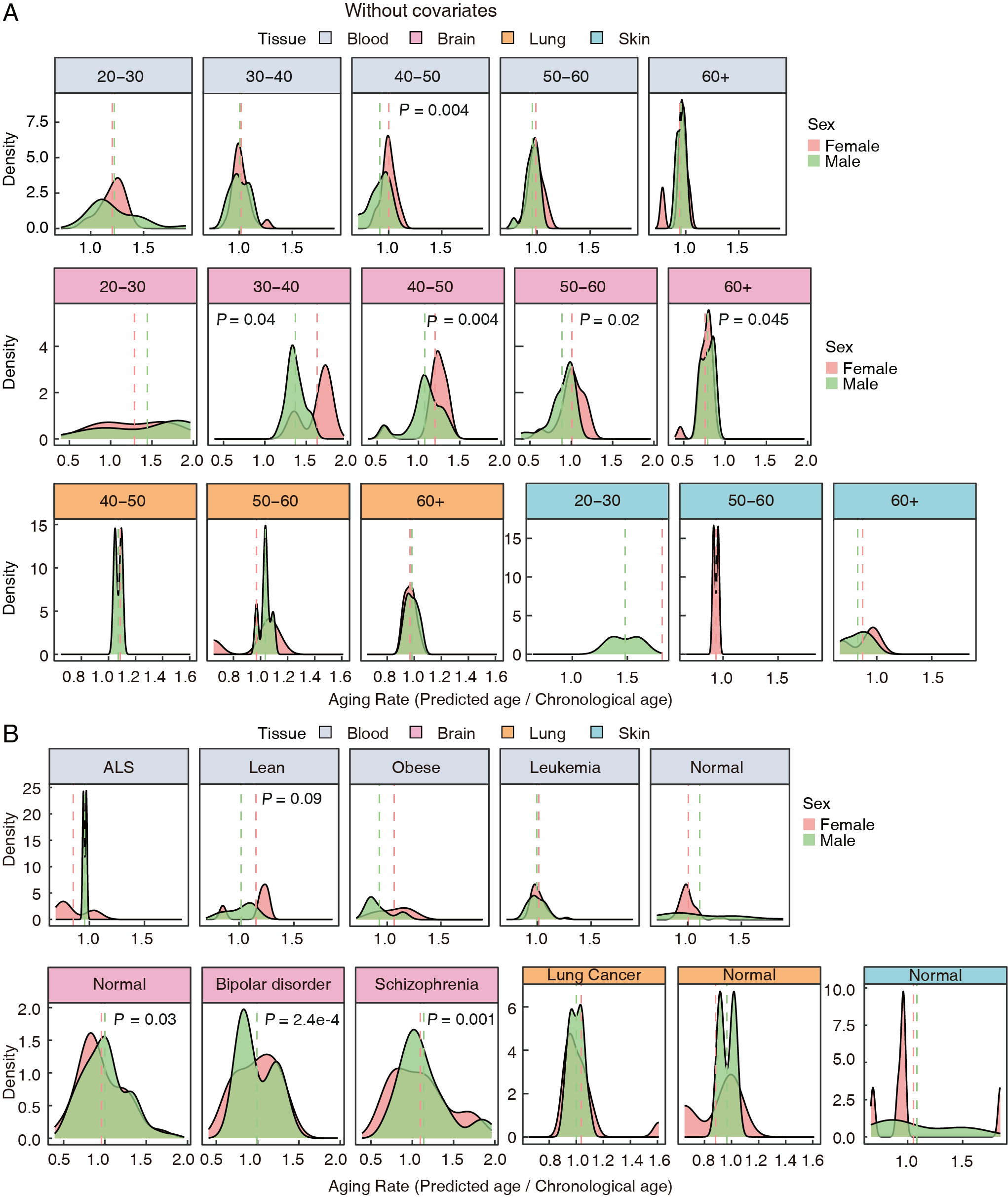


**Supplementary Figure 4. Associations between clinical phenotypes and aging using a model without disease status as a covariate.** (A) Differences in aging rates between males and females at different age ranges in four tissues. (B) Differences in aging rates between males and females under different disease states in four tissues.


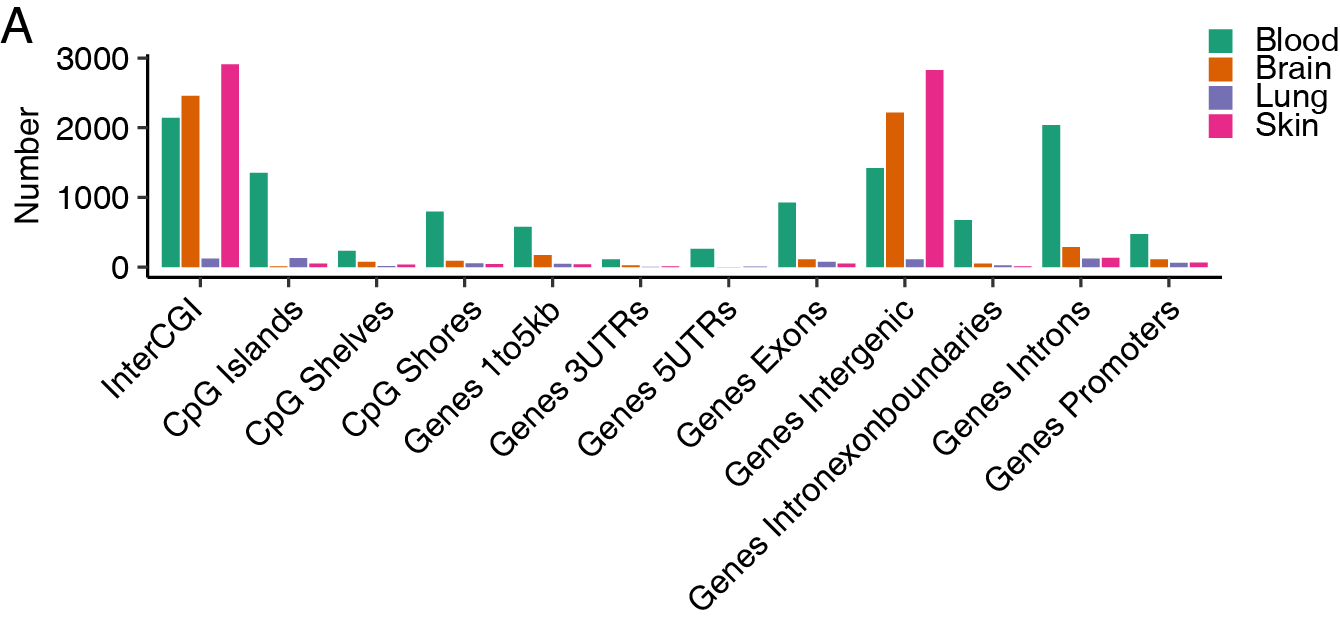


**Supplementary Figure 5.** **CpG and genic annotation of selected CpG sites.** (A) Bar plot showing the distribution of different annotation categories across the four tissues.


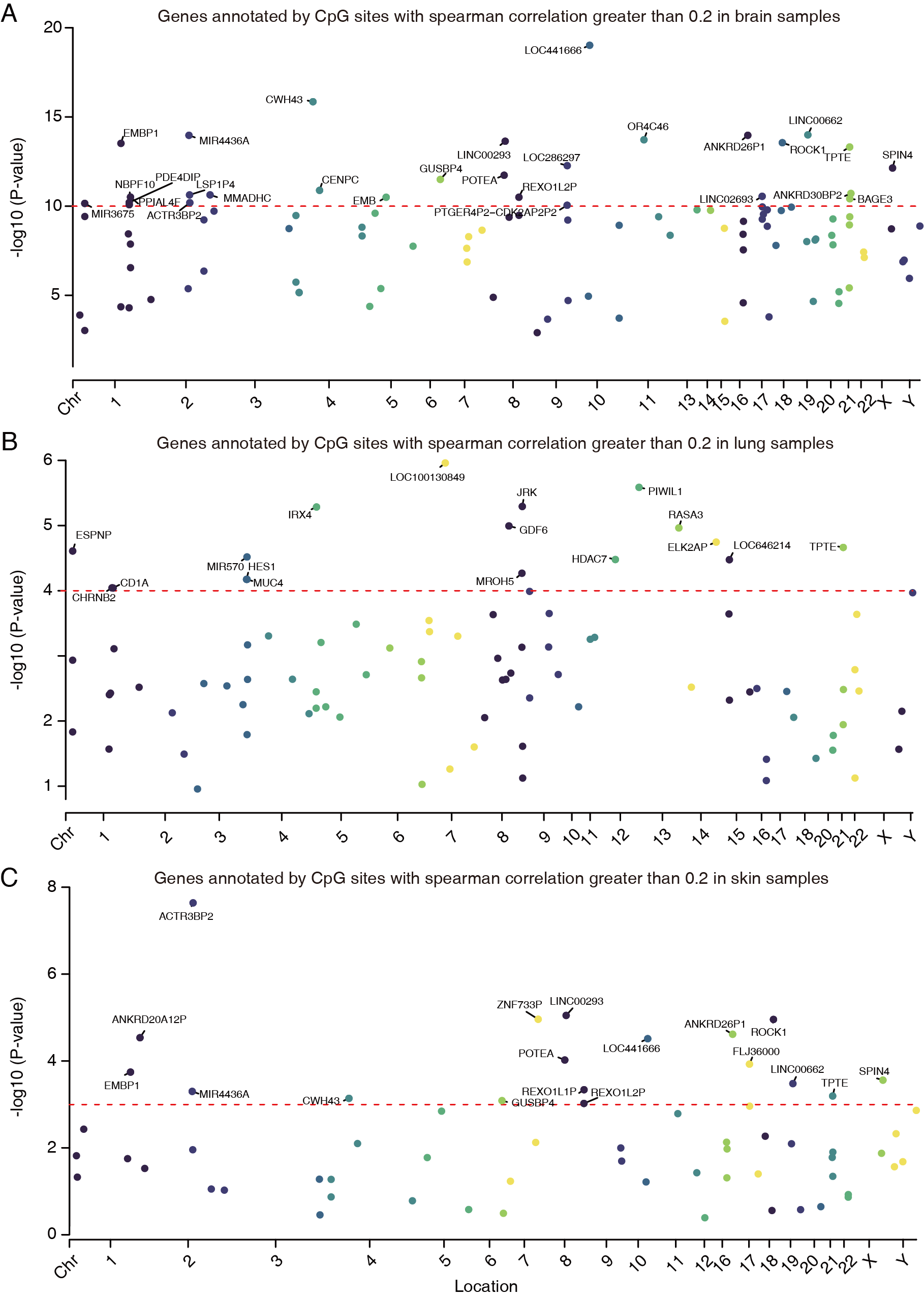


**Supplementary Figure 6.** **Manhattan plot showing the results of gene annotation.** (A-C) Manhattan plots for brain (A), lung (B), and skin (C) tissues displaying *P*-values of selected genes by BS-clock using a correlation test, highlighting significant genes with *P* values ​​below 1e-10, 1e-10 and 1e-3, respectively.


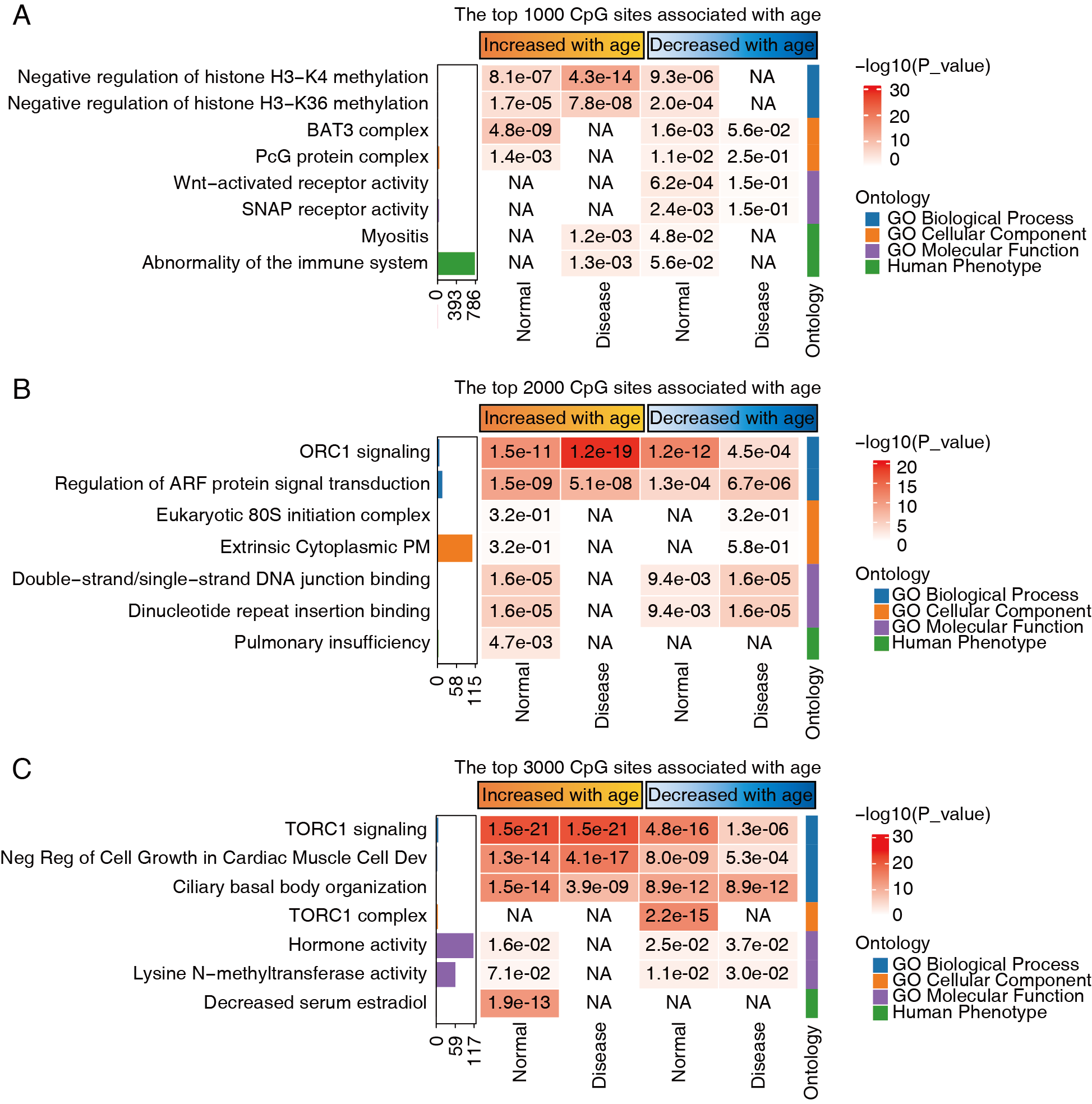


**Supplementary Figure 7.** **GREAT functional enrichment analysis of selected CpG site features.** (A-C) GREAT functional enrichment analysis based on the top 1000 (A), 2000 (B), and 3000 (C) CpGs that increase or decrease with age in normal and disease samples. The y-axis lists the name of a functional gene sets/biological pathways, sorted by ontology and the most significant *P*-value within each ontology. The bar plots in the first column report the total number of genes at each studied gene set. The heatmap color codes -log10 (*P*-value).


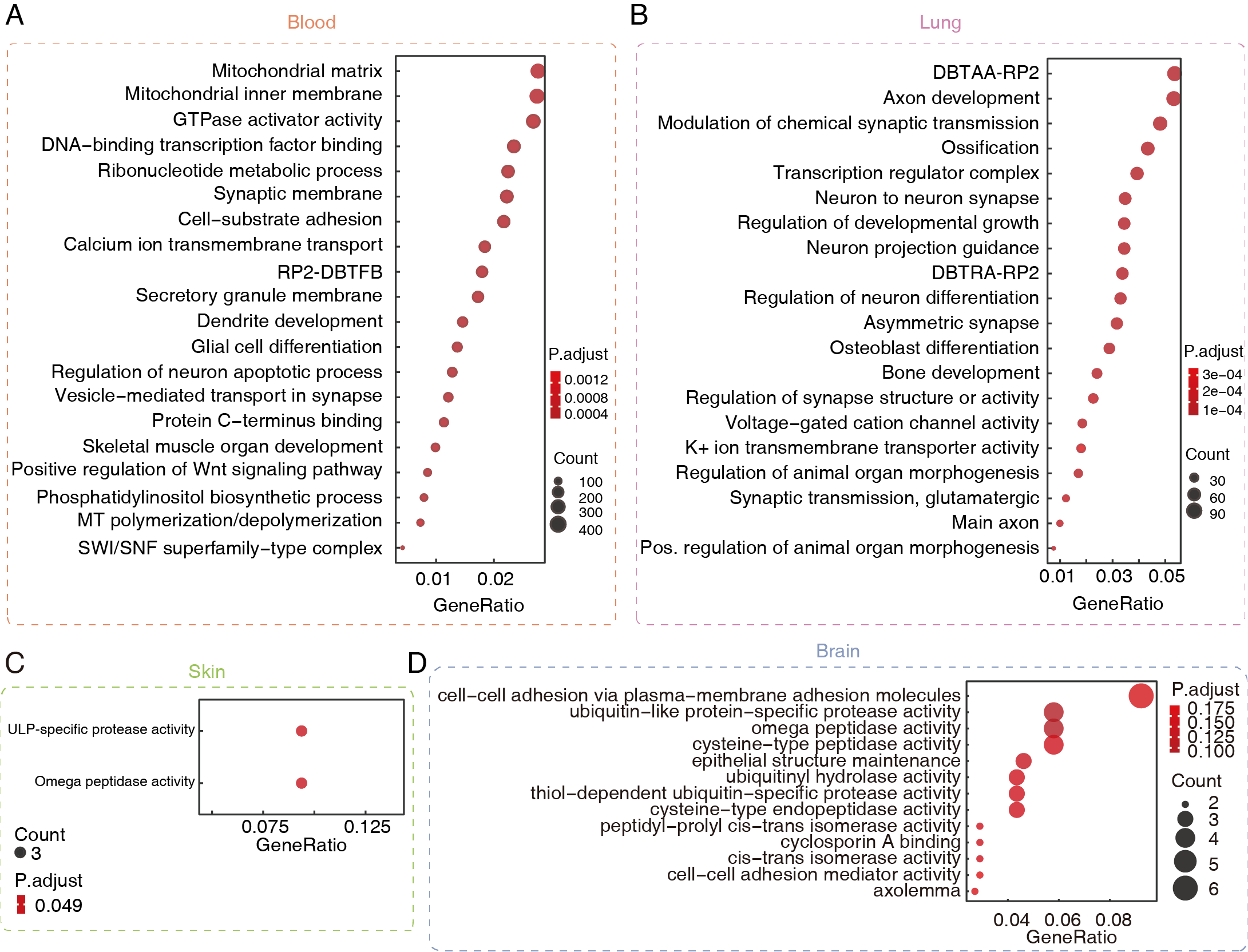


**Supplementary Figure 8. GO enrichment analysis of tissue-specific CpG site features.** (A-C) GO enrichment analysis of CpG sites unique to blood (A), lung (B), skin (C), and brain (D) tissues.
