# Supplementary figures and images for: BS-clock, advancing epigenetic age prediction with high-resolution DNA methylation bisulfite sequencing data
Source: Bioinformatics. 2024 Nov 5;40(11):btae656. doi: 10.1093/bioinformatics/btae656 (PMC11572488; doi:10.1093/bioinformatics/btae656)

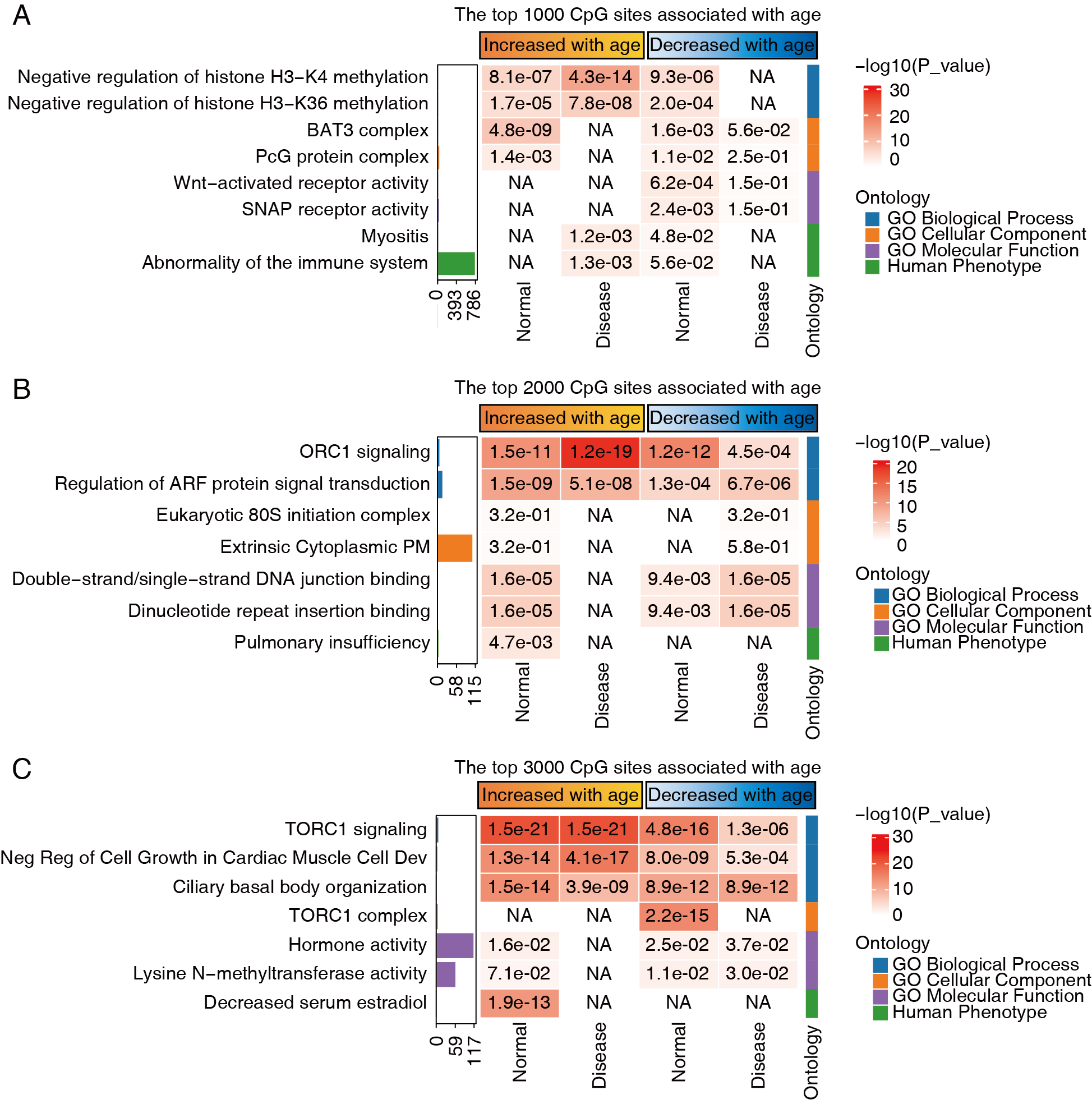

Supplement: btae656_Supplementary_Data [file btae656_supplementary_data.zip › Supplementary figure7.png]

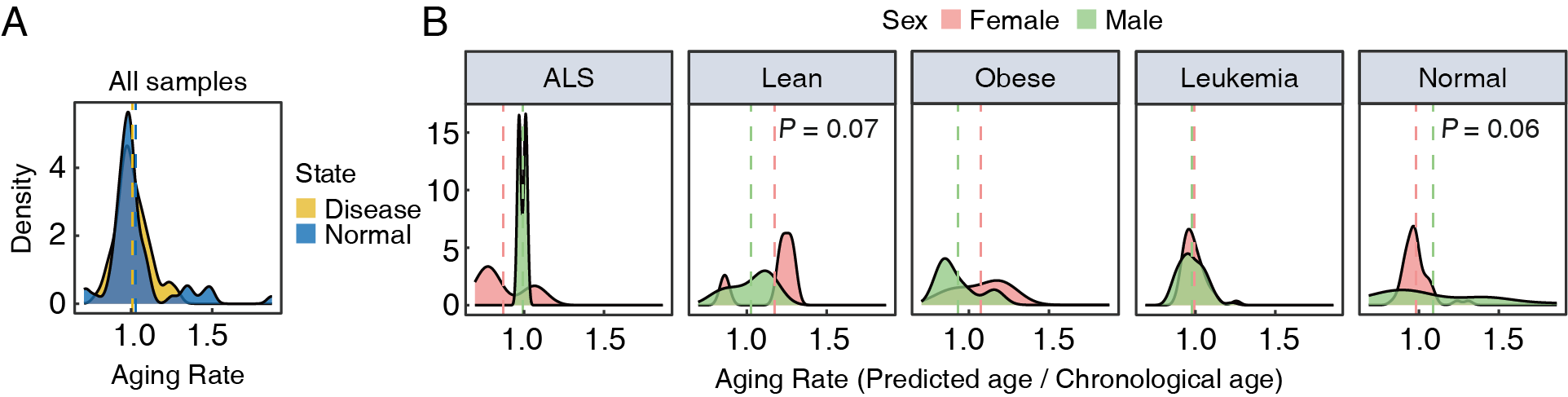

Supplement: btae656_Supplementary_Data [file btae656_supplementary_data.zip › Supplementary figure2.png]

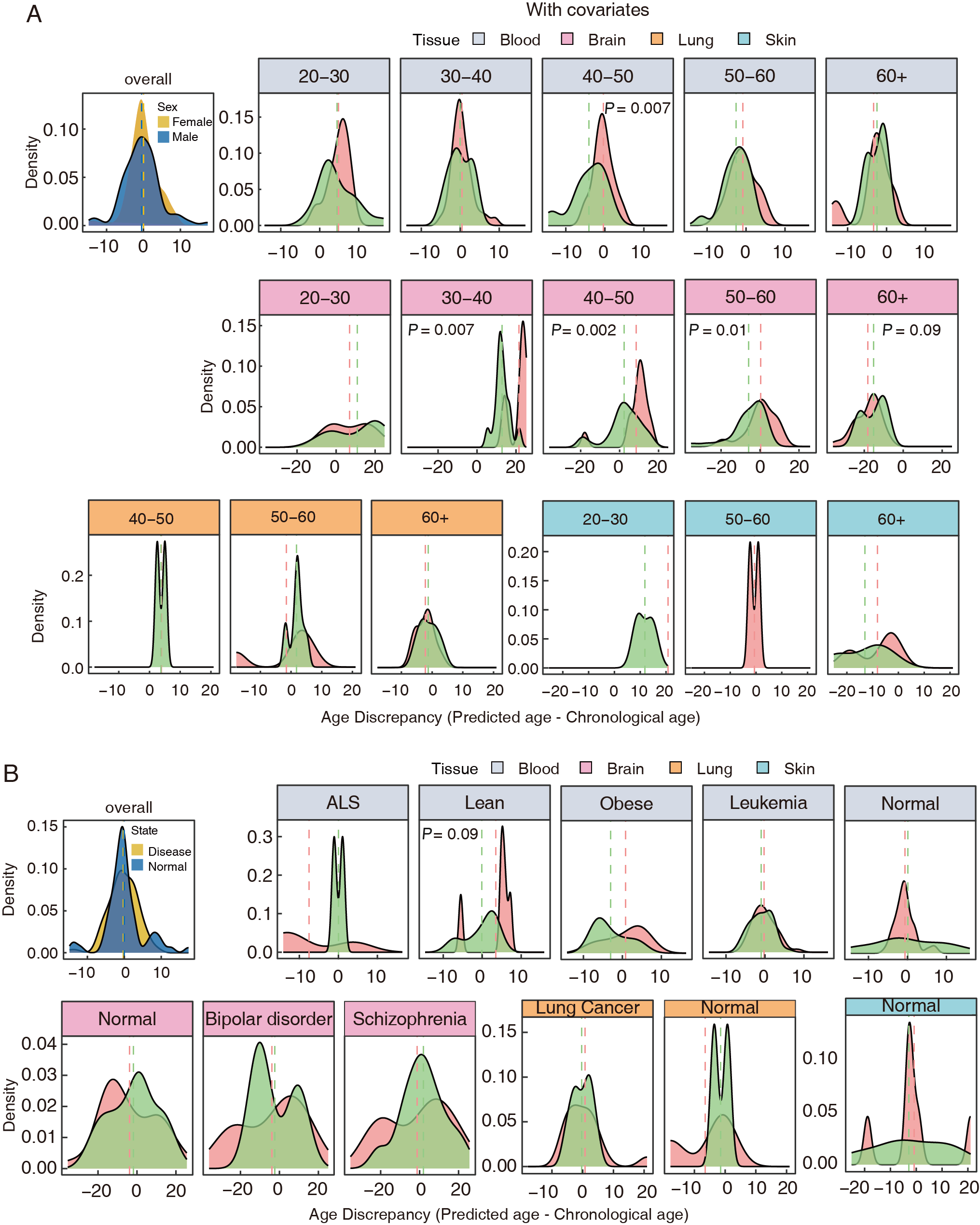

Supplement: btae656_Supplementary_Data [file btae656_supplementary_data.zip › Supplementary figure3.png]

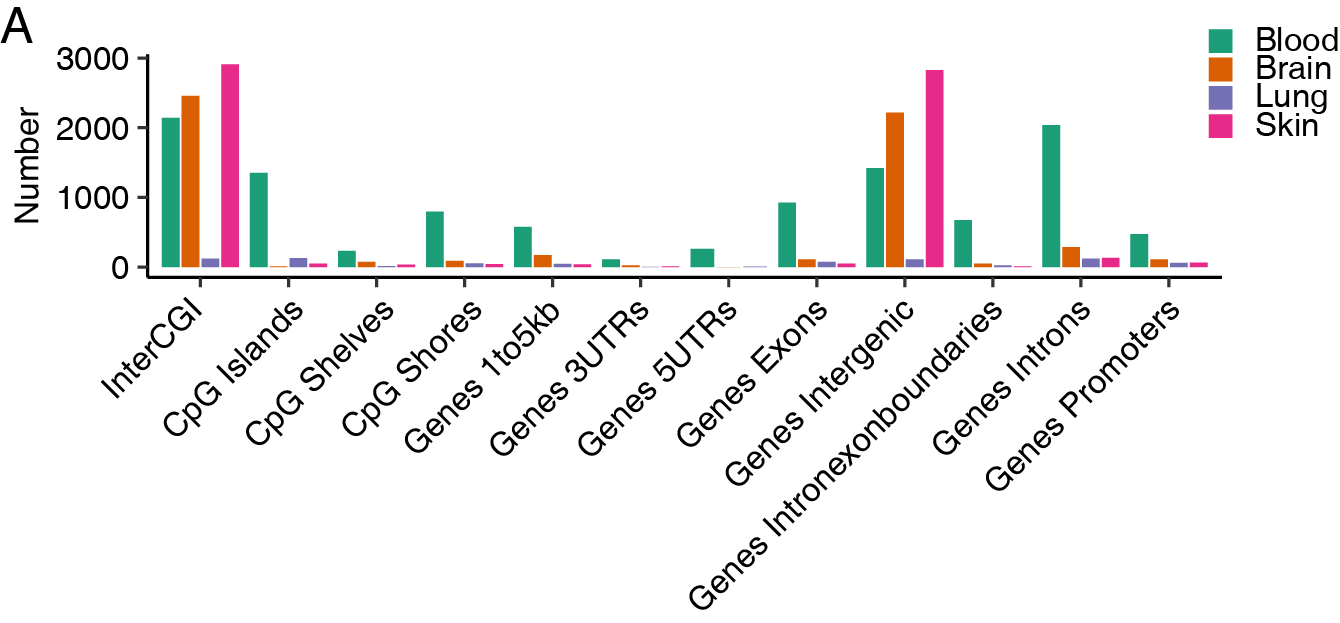

Supplement: btae656_Supplementary_Data [file btae656_supplementary_data.zip › Supplementary figure5.png]

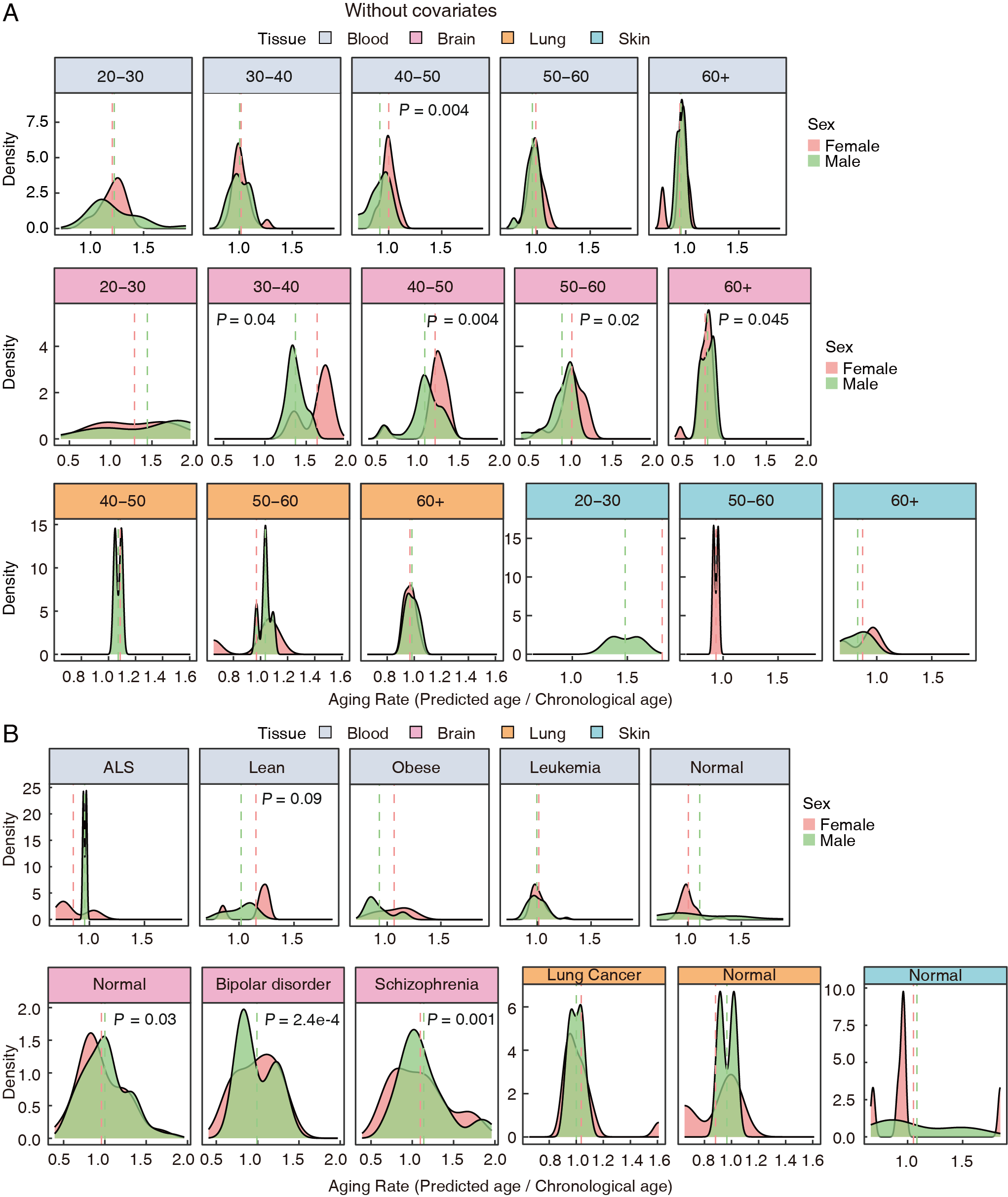

Supplement: btae656_Supplementary_Data [file btae656_supplementary_data.zip › Supplementary figure4.png]

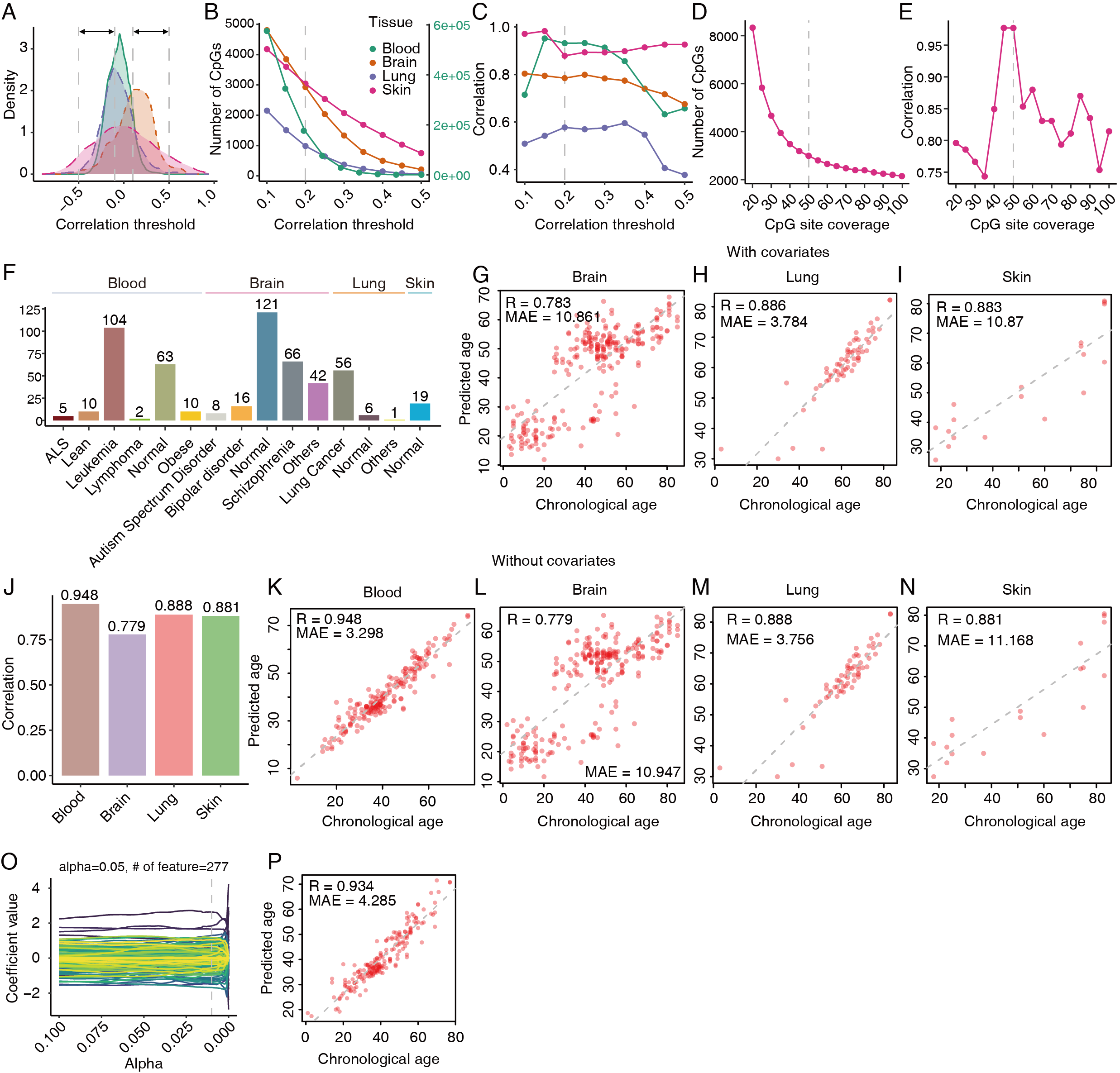

Supplement: btae656_Supplementary_Data [file btae656_supplementary_data.zip › Supplementary Figure1.png]

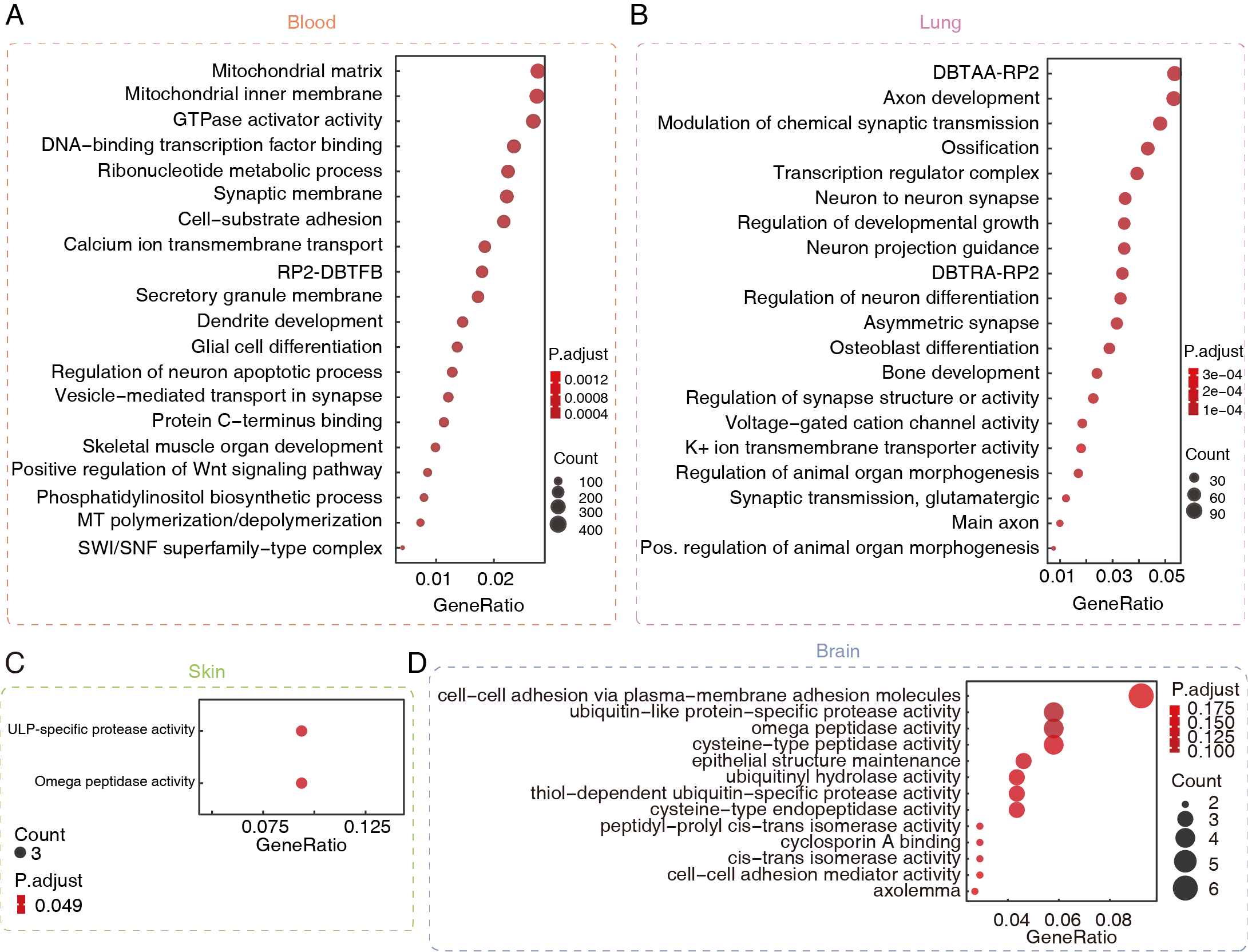

Supplement: btae656_Supplementary_Data [file btae656_supplementary_data.zip › Supplementary figure8.png]

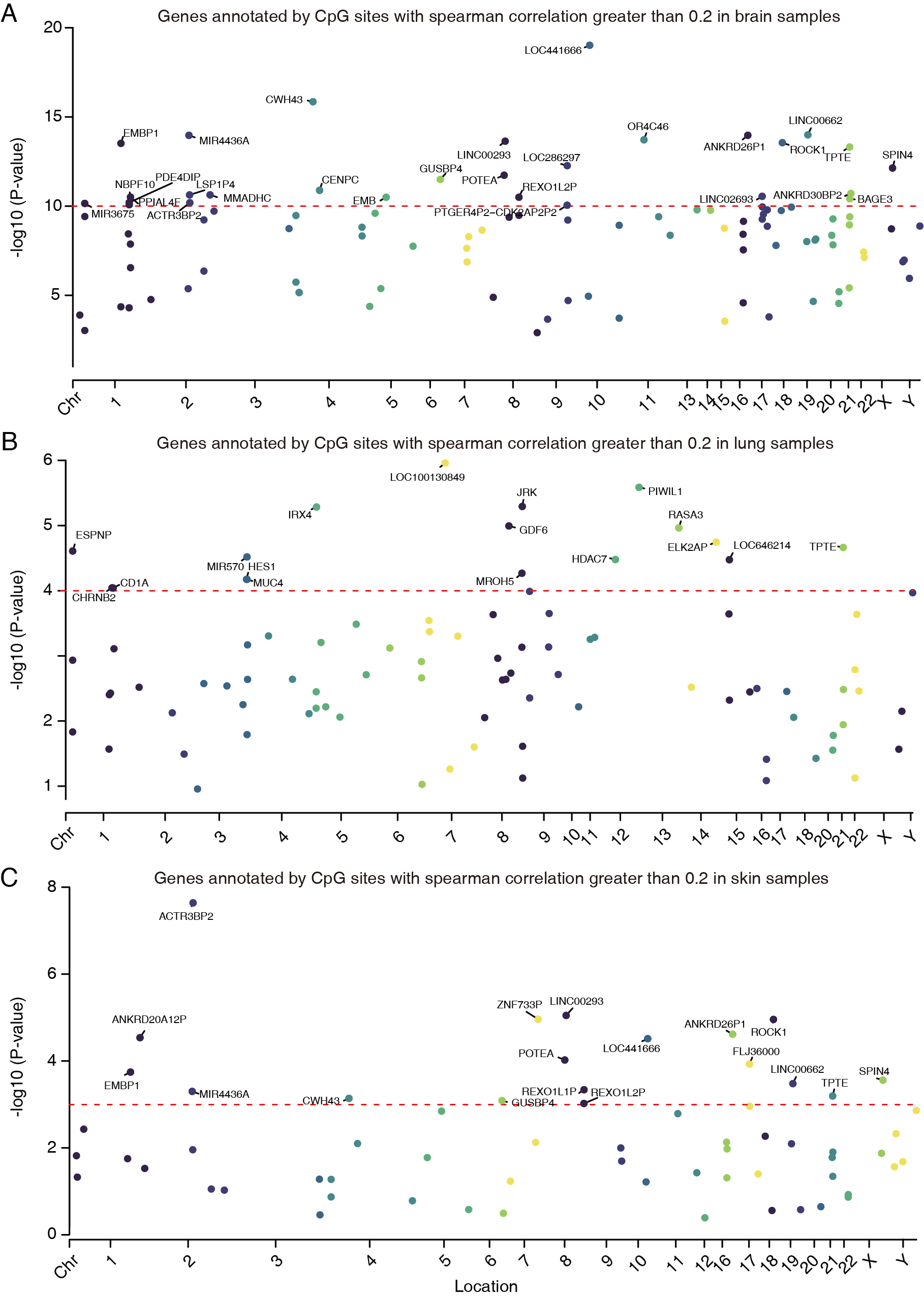

Supplement: btae656_Supplementary_Data [file btae656_supplementary_data.zip › Supplementary figure6.png]
